# An expanding human footprint drives escalating human–elephant conflict across a transboundary African landscape through 2085

**DOI:** 10.1093/pnasnexus/pgag205

**Published:** 2026-07-07

**Authors:** Evan Patrick, Maxwell Pepperdine, Christy Yu, Sophie Pesek, Olivia Somhegyi, Joana M Krieger, Nickolas McManus, Ezequiel Fabiano, Colgar Sikopo, Patrick R Roehrdanz, Ashley Larsen

**Affiliations:** Bren School of Environmental Science and Management, University of California, Santa Barbara, CA 93106-5131, USA; Bren School of Environmental Science and Management, University of California, Santa Barbara, CA 93106-5131, USA; Bren School of Environmental Science and Management, University of California, Santa Barbara, CA 93106-5131, USA; Energy and Resources Group, University of California, Berkeley, Berkeley, CA 94720, USA; Conservation Biology Institute, Corvallis, OR 97333, USA; The Moore Center for Science and Solutions, Conservation International, Arlington, VA 22202, USA; The Moore Center for Science and Solutions, Conservation International, Arlington, VA 22202, USA; Department of Wildlife Management and Tourism Studies, University of Namibia, Katima Mulilo 1096, Namibia; Ministry of Environment, Forestry and Tourism, Windhoek 13306, Namibia; The Moore Center for Science and Solutions, Conservation International, Arlington, VA 22202, USA; Bren School of Environmental Science and Management, University of California, Santa Barbara, CA 93106-5131, USA

**Keywords:** human–wildlife conflict, land-use change, biodiversity, causal inference, species distribution modeling

## Abstract

Rapid land-use change is compressing the space between people and wildlife at an unprecedented pace, elevating concerns of conflict between people and keystone megafauna such as the African savanna elephant (*Loxodonta africana*). However, the underlying drivers of human–elephant conflict (HEC) are often inferred but not causally identified, and forecasting under future scenarios is limited, restricting our ability to anticipate where and why conflict will intensify. Using a dataset on conflict events from 2004 to 2020 across a transboundary landscape in Southern Africa, we integrate causal inference (panel data regressions) and machine learning (point process models) to link mechanisms with spatial forecasts. We find that human population growth, cropland expansion, and climate-driven aridity in core elephant-protected areas drive conflict risk. Furthermore, we show that landscape features such as wildlife fences and roads constrain elephant movement in ways that drive HEC. Applying these models to projected land use, population, and climate under future coupled climate-development scenarios, we find that the area at high risk of HEC increases by 33 to 100% by 2085 with corresponding rises in event frequency. Aggressive human land-use expansion leads to the most dramatic increases in conflict, with climate impacts playing a significant but smaller role. These results identify emerging conflict hotspots decades in advance, offering actionable foresight for land-use planning and mitigation in a region critical to elephant conservation.

Significance statement Human–wildlife conflict is accelerating globally as agriculture and settlements expand into wildlife ranges, yet planners lack spatially explicit tools to anticipate where conflict will intensify. Using a multimodel approach, we identify the drivers and spatial patterns of historic and future elephant crop raiding across Africa's largest transboundary conservation landscape. We find human land use and population expansion primarily drive conflict rates, outweighing impacts from climate, and pinpoint locations where conflict risk will intensify decades in advance, providing an unprecedented window for intervention. By combining two complementary forecasting methods, we create a more complete picture of potential shifts in the patterns of human–wildlife conflict, offering a framework for researchers to assess conflict risk and future change in transfrontier landscapes globally.

## Introduction

Global environmental change is reshaping interactions between people and wildlife. Expansion of agriculture, settlements, and infrastructure is increasing the frequency of encounters, while climate-driven shifts in water and forage availability can redirect animal movement toward human resources and amplify conflict (1). Recent global analyses project that human–wildlife overlap will expand across more than half of terrestrial land area by 2070 (2), heightening the need for spatially explicit tools that can anticipate emerging conflict hotspots and identify actionable drivers.

Southern Africa supports over 290,000 savanna elephants, accounting for roughly 70% of Africa's savanna elephants, with many populations stabilizing or rebounding in recent decades (3). At the same time, the region's human population is growing at ∼2.5% annually (4), leading to the expansion of human settlements and agriculture and increasing spillovers of elephants into human-dominated landscapes. Resulting human–elephant conflict (HEC), including crop raiding, injury to people, or destruction of infrastructure and livestock, is often devastating for impacted households and can erode community support of conservation (5, 6). In extreme cases, this leads to the culling of elephants (7), underscoring a consequential misalignment between conservation priorities and human livelihood policies. These trends, alongside the potential of growing climate pressures to further escalate conflict (8), present critical challenges for resource managers in the region.

These conservation challenges intersect directly with the socioeconomic realities of affected communities. In Namibia, elephant crop raiding, the most common form of HEC, can result in economic damages that outweigh local benefits from trophy hunting (9). Given its clear relevance to conservation and livelihoods, many studies have examined the drivers and distribution of HEC (10–13). However, most of these studies do not consider global change pathways that could substantially alter elephant movement, population health, and conflict prevalence in the future (14, 15). While past work has shown overlap between human-dominated landscapes and elephant ranges under varying climate scenarios (15) and climate is recognized as a conflict amplifier (1), there remains a critical need to map the drivers, prevalence, and extent of HEC into the future.

Previous research on HEC has generally used one of two approaches based on disciplinary focus: either causal inference statistical methods or machine learning models. Causal inference methods, common in economics, political science, and public health, identify specific drivers of HEC, such as settlement density (16) or cropping patterns (17). These methods help describe the mechanisms that give rise to HEC, though they are sometimes challenged by complex, nonlinear and dynamic ecological relationships. Alternatively, a growing body of HEC research applies methods derived from machine learning to understand the patterns and distribution of HEC (18, 19), providing explicit maps of conflict hotspots with generally higher predictive power than regression methods, though with less focus on mechanisms or causality. In our analysis, we integrate these complementary approaches, leveraging the causal identification strengths of econometric methods and the spatial predictive power of machine learning to assess underlying causal drivers and spatial hotspots of HEC now and under future scenarios.

**Figure 1:**
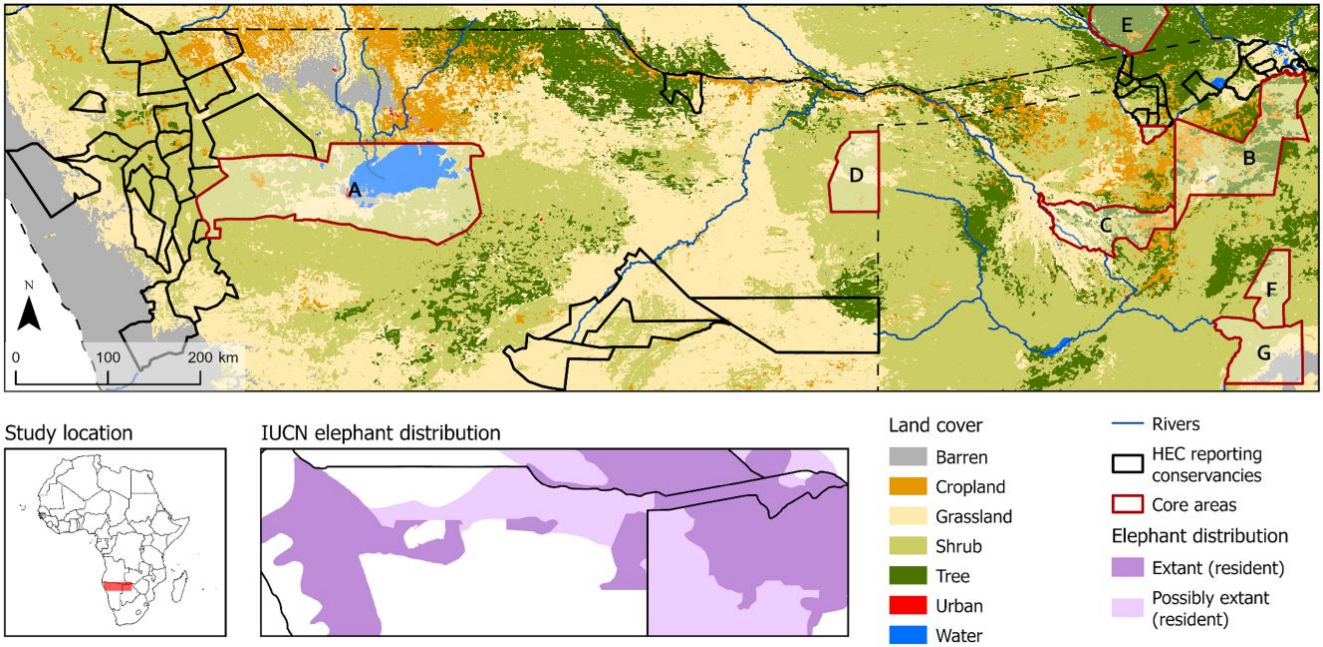
Study area extent. Land-cover data are from Chen et al. (20), showing land-cover types in 2015. Rivers are from the HydroRivers dataset defined as greater than ord_flow 5 (10 km^2^ or an average river flow of at least 0.1 m^3^/s). Core area boundaries were sourced from the WDPA dataset (21) and identified from (3). They are A) Etosha National Park, B) Chobe, C) Moremi, D) Khaudum, E) Sioma Ngwezi, F) Nxai Pan, and G) Makgadikgadi Pans. The core area adjacent to the top left corner of Chobe (B) is Nkasa Rupara.

Using a database that tracked incidents of human–wildlife conflict, including HEC, from 2004 to 2020 across Namibia's communal conservancies (community-managed reserves that support tourism and sustainable resource use), we analyzed the drivers and spatial distribution of elephant crop raiding across a transboundary landscape spanning northern Namibia, much of northern Botswana, and portions of Angola and Zambia (Fig. 1), hereafter referred to as the study area. We then projected both models forward under multiple coupled climate-development scenarios (Shared Socioeconomic Pathway–Representative Concentration Pathway [SSP–RCPs]) to produce spatially explicit estimates of changes in the frequency and extent of HEC through 2085. This integration of causal and machine learning approaches allowed us to ask the following questions: (i) What are the current drivers of HEC in the study area? (ii) What are the predicted spatial hotspots of HEC events across our study area? and (iii) How will the spatial extent and frequency of HEC change under divergent climate and development pathways? In answering these questions, we provide key insights to guide proactive HEC management in the decades ahead.

## Methods

### Study area

The study area spans northern Namibia, much of northern Botswana, and portions of Angola and Zambia, encompassing Namibian communal conservancies where HEC was recorded and the surrounding landscapes used by elephants (Fig. 1). Elephant populations extend across the north of the study area, with the largest population spanning the transfrontier region of northeast Namibia and adjacent countries (22), a population within the fully fenced Etosha National Park, and a smaller desert-adapted population in Namibia's northwestern highlands (23). HEC is most prevalent in the far eastern Zambezi Region and the northwestern highlands, where elephant ranges overlap or border rural human settlements. The Zambezi Region, located within Namibia's eastern panhandle, stands out within Namibia as being relatively wet and amenable to farming, leading to a higher human population density and agricultural footprint than the rest of the country (24). It also serves as a functional corridor between core elephant reserves, such as Chobe and Sioma Ngwezi National Parks (22). Namibia's northwestern highlands experience low, variable rainfall and erosive soil, leading to heterogeneous resource availability and highly mobile resource searching behavior in elephants (25). Across the study area, climate is strongly seasonal, with most rainfall falling during the wet season from roughly November to April and a pronounced dry season from May to October, although rainfall totals generally decline from the wetter eastern portion of the study area toward the more arid northwestern Namibian highlands (26).

We focused on HEC events recorded within Namibia's communal conservancies, but our study area considered the broader landscapes surrounding these conservancies in evaluating the drivers and distribution of HEC. Communal conservancies are self-governing, communal land management organizations formed to gain common property rights over wildlife and tourism operations on local lands (27). While they utilize land-use restrictions to promote conservation, subsistence farming remains a key livelihood strategy for residents. We assess HEC across 38 communal conservancies, with a combined population of 147,103 people (28) and a rapid growth rate (Fig. S1).

### Modeling approach

All model fitting was performed on historical HEC and covariate data from 2004 to 2020. We fit two complementary model classes to these data: two-way fixed-effects panel regressions to estimate changes in crop-raiding counts and season-specific point process models to estimate spatial HEC risk. We first report the outputs of these historically fitted models, and then apply them, without refitting, to projected future climate, land cover, and population data to generate out-of-sample projections of future conflict frequency and distribution. Later sections provide the detailed data sources and model specifications.

### HEC data

We obtained monthly HEC data for 2004–2020 from the Conservation Information (ConInfo) database managed by the Natural Resources Working Group of the Namibian Association of Community Based Natural Resource Management. Events were self-reported by farmers and verified by audits as part of a compensation payment program (29). Data were recorded in a 2 km grid (Fig. S7) that serves as our unit of analysis (hereafter referred to as the “HEC grid”). We focused on crop-raiding events, which constituted over 90% of the conflict events in the dataset. As crop raiding is likely influenced by seasonal changes in vegetation productivity (10, 30), events were aggregated by wet (November–April) and dry (May–October) seasons. May events were included in the wet season to account for crop harvesting that extends into the early dry season.

### Landscape characteristics

At the landscape level, we compiled datasets describing human landscape use, features that elephants preferentially use, and features that may hinder elephant movement. Because HEC was recorded on a fixed 2-km grid, all predictors were aligned to this grid before analysis: continuous rasters were aggregated by mean, land-cover classes were converted to percent cover within each cell, vector features were transformed to distance-to-feature rasters prior to aggregation, and population counts were averaged from a high-resolution population surface to the HEC grid. These choices aim to conserve the specificity of HEC predictors while still conforming to the 2-km resolution at which events were recorded.

Since roads can act as either barriers or corridors for elephant movement and are associated with HEC events (18), we included distance to roads as a predictor variable, using road data from OpenStreetMap (31). We pulled yearly human population counts from WorldPop (32, 33), as population is a well-documented predictor of HEC (12, 16, 17). Annual percentage-cover estimates for built-up area, cropland, shrub cover, grassland cover, tree cover, and water were extracted for each 2-km HEC grid cell for 2004–2020 from the Climate Change Initiative (CCI) Global Plant Functional Type dataset (34). These year-specific land-cover values were used as time-varying covariates in the historical analyses. Because elephants often track anomalously green patches across the landscape (30), we used season-year mean Enhanced Vegetation Index (EVI) from Terra MODIS MOD13Q1 V061 (16 day, 250 m), an atmospherically corrected, quality-screened composite product, for 2004–2020 (35). We selected EVI as a standardized landscape-wide greenness metric for this heterogeneous study area; although soil-adjusted indices such as SAVI can be advantageous in sparsely vegetated settings with strong soil background, EVI provided a consistent measure of seasonal vegetation productivity for the region.

Water sources are also important preferential features for elephant movement (36); therefore, we used global rivers data from HydroRIVERS, filtered to rivers with catchment areas of at least 10 km^2^ or an average river flow of at least 0.1 m^3^/s, or both (37), and calculated distance to the nearest river. Veterinary fences, which can shift conflict by concentrating elephant movement (38), were taken from Huang et al. (22) to calculate distance to the nearest fence. Elevation and slope were derived from US Geological Survey (39) to characterize topographic variability. Finally, to assess connectivity from HEC grid cells to elephant population centers, we used elephant core areas defined by Huang et al. (3) sourced from Protected Planet (21) and calculated distance to the nearest core area.

### Historical climate data

For 2004–2020, we extracted monthly precipitation and mean temperature from the CHELSA dataset at 30 arc-second (∼1 km) resolution (40), resampled to the 2 km conflict grid using mean aggregation, and used these to calculate monthly 3-month Standardized Precipitation Evapotranspiration Index (SPEI-3) values. When CHELSA data were unavailable (mainly after June 2019), we used ERA5-Land surface temperature and summed precipitation at a 9-km resolution (41), resampled to the HEC grid using nearest neighbors. SPEI values were also computed for elephant core areas and for a 50-km buffer around each HEC grid cell to assess landscape-scale water availability. SPEI-3 is a rolling monthly index, such that each monthly value reflects climate conditions in the focal month and the previous 2 months. We then averaged monthly SPEI-3 values within each season year used in the analysis.

### Global change datasets

To project future HEC dynamics, we extracted 3-month SPEI from the Global Drought Layers dataset, which provides 0.25° projected drought indices from 1980 to 2100 based on downscaled Climate Model Intercomparison Project Phase 6 models across SSPs (42). We used the five models identified in the Intersectoral Impact Model Intercomparison Project as per (40) and calculated mean wet- and dry-season 3-month SPEI under RCP 2.6, RCP4.5, RCP7.0, and RCP8.5 for 2041–2070 and 2071–2100. These values represent the average SPEI departure across models from the 1980–2015 historical baseline to 2055 and 2085, the midpoints of these 30-year ranges.

Future land cover was taken from global projections by (20), which provide 1 km resolution classifications every 5 years for 2015–2100 under each coupled SSP–RCP scenario. We extracted the same cover types used in the historical CCI dataset and converted categorical data to percentage cover at the HEC grid resolution, producing coarse percentage estimates roughly consistent with the historical baseline. Human population density for 2025, 2055, and 2085 was obtained from the FPOP dataset (43), which provides 1 km global projections for SSPs 1–5 from 2020 to 2100. These were used to calculate population change at the HEC grid level.

### Panel data regressions

We used two-way fixed-effects regression models to identify causal effects of land use and climate variables on HEC. By including fixed effects for spatial groups (500 *k*-nearest neighbors) and season years, we exploit within-group temporal variation while removing time-invariant unobserved heterogeneity that could bias estimates in complex ecological systems. This approach differentiates unobserved characteristics at the spatial group level and accounts for temporal shocks affecting all conservancies. We verified that our treatment variables, including weather, population, and land use, retained sufficient within-group variation (>10%) for identification (44). While retaining 10% of the total variation within each group, we spatially define 500 *k*-nearest neighbor groups to control for variation across the landscape and season-year groups to control for variation across time. SEs were clustered at both the spatial group and season-year levels following (45). The model specification is


(1) $$CropRaid_{igsy\;}=\beta \;X_{igsy}\;+\alpha_{g}\;+\;\gamma_{sy}\;+\;\epsilon_{igsy},$$

where *CropRaid_igsy_* represents the number of crop-raiding events at grid cell *i*, within spatial group *g*, in season year *sy.*. $X_{igsy}$ is a vector of time-varying covariates (population, land cover, and climate variables) with coefficient vector *β*. $\alpha_{g}$ denotes spatial group fixed effects (based on 500 *k*-nearest neighbors), $\gamma_{sy}$ represents season-year fixed effects, and $\epsilon_{igsy}$ is the error term. Summary statistics of the range and mean values of the events are provided in Table S1.

Finally, because conflict data were provided in count form, we ran negative binomial and Poisson fixed-effects regressions to ensure that our linear specification was not overestimating certain results. These models are discussed in Text S1, and the results are presented in Tables S2 and S3.

### Point process model

A point process modeling approach was used to predict the probability of HEC across the study area as a function of key predictor variables. Point process models are commonly used in species distribution modeling to predict distribution based on presence-only records and environmental predictor variables (46). While frequently applied to species occurrence distribution, point process models have also been employed to model the occurrence distribution of HEC (11, 18, 19). We generated two discrete point process models to separately explore patterns and changes in crop raiding during the wet versus the dry season. Occurrences were split by wet/dry season due to strong previously documented patterns and seasonality of HEC (12, 16, 17). For each model, the final output was a probability distribution across the study area defined by a bounding box encompassing all HEC-reporting Namibian communal conservancies (Fig. S2). This is the same extent shown in Fig. 1.

While known absences are desired in species distribution and ecological niche modeling, this data is often unavailable (47). To account for this within each season, background pseudo-absence points were randomly generated using the “dismo” R package (version 1.3.14). These points were temporally distributed proportional to the number of HEC occurrences in each season year (e.g. wet season 2004) and within conservancy boundaries, during years in which HEC was reported (Table S5). We aggregated incidents of HEC at each grid cell for each wet and dry season annually between 2004 and 2020, from which we then extracted the conditions of environmental predictor variables to train the point process models.

Sixteen environmental predictor variables were selected based on their ecological importance to model HEC risk (11, 16, 18, 19): percent land cover (built, cropland, grassland, shrub, tree, and water), distance to protected areas, distance to rivers, distance to roads, distance to fences, population density, EVI, SPEI, elevation, slope, and aspect. We tested for variable correlation using Pearson's correlation coefficient (Fig. S4) as per (48).

We used the “ENMeval” R package (version 2.0.4) to generate and evaluate a series of point process models before selecting the “best”-performing model. Two feature class combinations, linear (L) and linear + quadratic (LQ), were used, with regularization multiplier values ranging from 0.5 to 3 in increments of 0.5, to generate a total of 12 models. More complex feature classes (product, hinge, and threshold) were excluded to avoid overfitting and because these relationships are not easily justified given known elephant movement ecology (47, 49). Spatial block (4-fold) cross-validation was used to partition the data for training and testing during model evaluation to control for spatial autocorrelation (49). A discussion of model performance evaluation is provided in Text S2. After selecting the best-performing model, the Jackknife test was used to evaluate the relative importance of each explanatory variable in predicting HEC when generating the final wet and dry season point process models.

### Future projections

After fitting the regression and point process models to historical data, we projected future HEC using projections of climate, land use, and population under four paired SSP–RCP scenarios: SSP1-RCP2.6 (“sustainability”), SSP2-RCP4.5 (“middle of the road”), SSP3-RCP7.0 (“regional rivalry”), and SSP5-RCP8.5 (“fossil-fueled development”). These coupled climate-development scenarios represent alternative futures that differ in future climate, human population trajectories, and land-cover change, allowing us to estimate potential future rates and spatial distributions of HEC under divergent pathways.

For temperature, precipitation, land cover, and population, we calculated changes from a baseline (2011–2040 climatology for climate data; 2025 for land cover and population) to projected periods (2041–2070/2055 and 2071–2100/2085). Features without projected estimates, such as fences, roads, and rivers, were held constant. Regression projections used the delta method to estimate SEs, applying the same covariate structure as the fitted models (50, 51). We report changes in HEC rates as 2085 rates subtracted from 2025 rates to ensure comparability within global change datasets. The best-performing point process model was rerun with future data to predict HEC distribution across the study area for 2025, 2055, and 2085, following best practices for species distribution modeling (47). Continuous point process model outputs were converted to binary presence/absence using the threshold (0.72) that maximized Cohen's Kappa for the point process model, and changes were mapped by subtracting the 2025 binary raster from 2055 and 2085 predictions. Thresholding enables the creation of simple, easily interpretable maps (47) to compare predicted hotspots in point process modeling.

## Results

### Seasonal distribution of crop raiding

Crop-raiding events were strongly seasonal across the study area. At the grid-cell level, wet-season counts ranged from 0 to 75 events (mean = 0.068), whereas dry-season counts ranged from 0 to 28 events (mean = 0.005; Table S1). Mean grid-cell crop-raiding counts were therefore more than an order of magnitude higher in the wet season, and wet-season counts also spanned a broader range of observed conflict. This contrast provides context for the season-specific analyses reported below.

### Drivers of HEC

We found that rising human population and increases in cropland and built-up cover led to higher levels of crop raiding, all else being equal. Population increases within fixed-effects groups were a significant predictor of crop raiding across seasons (Table 1), while increases in cropland and built area were associated with higher wet season conflict. We found that drier core elephant-protected areas, as measured through 3-month SPEI, were predictive of higher wet season conflict in nearby conservancies, suggesting a spillover effect from variable habitat suitability in reserves. While this climate driver was significant, variation in human population and land cover were overall more predictive of crop-raiding conflict over our study period.

**Table 1:** Fixed-effects regression estimates for crop-raiding models with SEs, clustered on spatial group and season year, in parentheses.

Scaled covariate	Crop-raiding model outputs	
	Wet season	Dry season
Grid spei3	−0.012 (0.016)	0.001 (0.001)
50 km spei3	0.014 (0.021)	−0.003 (0.002)
Core spei3	−0.081* (**0.037)**	0.003 (0.003)
Population	0.176*** (**0.028)**	0.011*** (**0.002)**
Tree cover	−0.019 (0.017)	−0.004 (0.003)
Cropland cover	0.053*** (**0.012)**	0.001 (0.001)
Built cover	0.022** (**0.006)**	0.001 (0.001)
Fixed effects		
Spatial group	Yes	Yes
Season year	Yes	Yes
Observations	126,304	126,304

Covariates were scaled to a consistent 0–1 range before running the regression, allowing for comparable coefficients within models. Bold values indicate statistically significant estimates and their corresponding SEs in parentheses. Grid spei3 is the 3-month SPEI calculated at the HEC grid level, 50 km spei3 represents 3-month SPEI values at a 50-km buffer around the HEC grid, and core spei3 is 3-month SPEI values in the nearest elephant core area. Population represents yearly human population counts sourced from WorldPop. Tree, cropland, and built cover are the percentage cover for each land-cover type, sourced from the Copernicus CCI Global Plant Functional Type dataset. *P < 0.05, **P < 0.01, and ***P < 0.001.

As event data often do not follow a normal distribution as required for unbiased linear models, we also ran Poisson and negative binomial regressions, which are tailored to the distributions expected from zero-bounded count data. These regressions produced similar results, confirming the robustness of anthropogenic predictors to count model specifications (Tables S2 and S3). We also estimated random effects models to incorporate time-invariant landscape characteristics into our prediction of HEC events. While Hausman tests indicated fixed-effects specifications were preferred for wet season crop raiding (*P* < 0.05), we found that areas closer to fences and farther from roads were more likely to experience conflict, suggesting that these features constrain elephant movement and may increase HEC risk (Table S4).

### Predicted conflict hotspots

**Figure 2:**
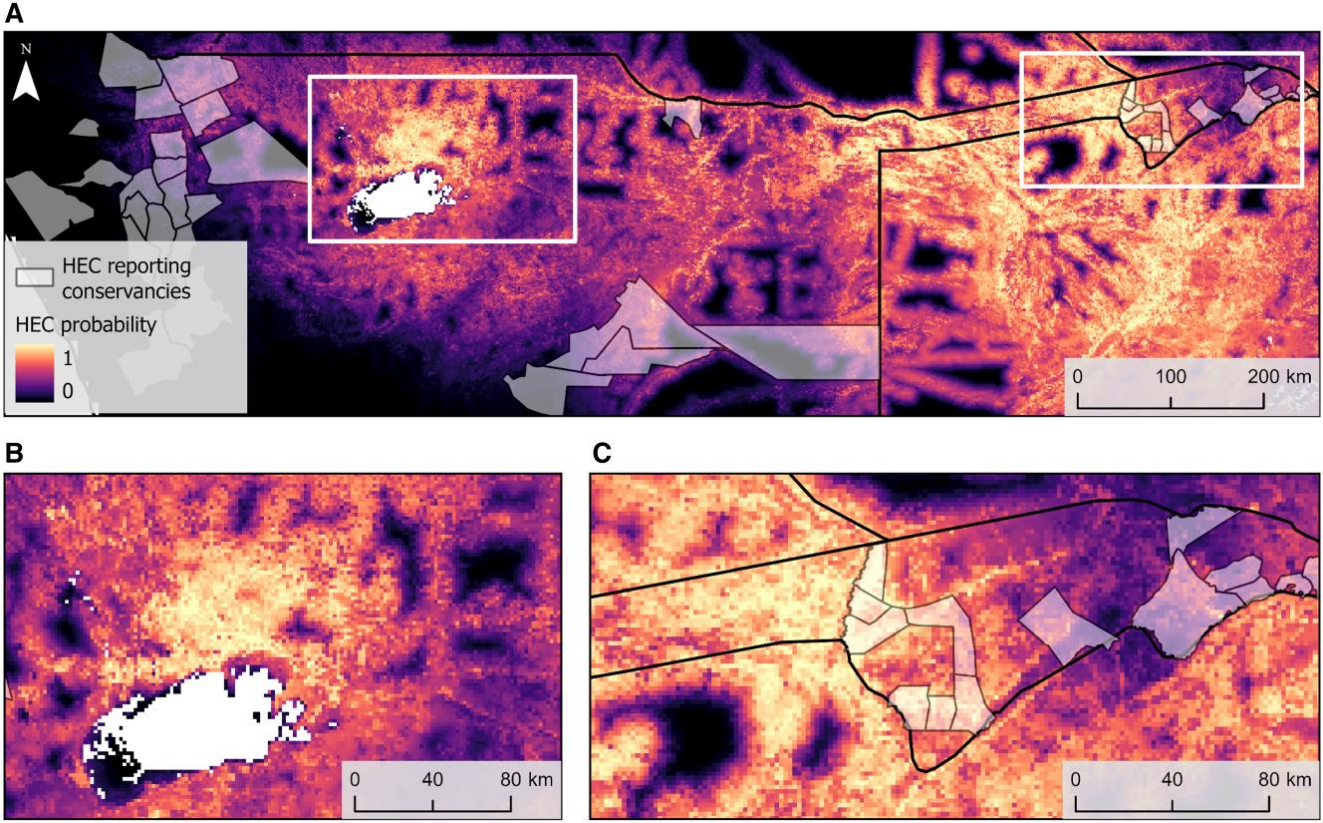
Baseline predictions for the probability of elephant crop raiding during the 2020 wet season. The entire extent of the point process model predictions (A) is the same extent as Fig. 1, generated by creating a bounding box (Fig. S2) around all HEC-reporting communal conservancies (white polygons). HEC-reporting conservancies are all Namibian communal conservancies that reported incidents of HEC in the conflict data set used in the analysis. Two predicted HEC risk hotspots are shown in the inset maps: regions north of Etosha National Park (B) in northwest Namibia and the Zambezi Region (C) in northeast Namibia. The white spot in B is Etosha Pan, a large salt pan covering almost a quarter of Etosha National Park.

Using a point process modeling approach, we generated predictions of continuous HEC probability throughout the study area (Fig. 1) as a function of key environmental predictor variables. During the wet season, predicted crop-raiding hotspots are concentrated along the northern boundary of Etosha National Park in northwest Namibia (Fig. 2B) and across the Zambezi Region in the northeast (Fig. 2C). Additional high-risk areas include the western section of Namibia's panhandle in the Kavango East Region and areas north of Khaudum National Park. In northern Botswana, much of the region included in the study area was also identified as a HEC hotspot, particularly regions along the Okavango Delta and near the Moremi Wildlife Reserve. Predictions of crop raiding during the dry season were broadly similar to those observed in the wet season (Fig. S3). Jackknife analyses of the point process models indicate that regularized training gains declined most when tree cover, distance to roads, distance to fences, population, and distance to rivers were excluded (Fig. S6), implying that these variables best contributed to the collective model's HEC predictive capability. Across both seasonal models, EVI and tree cover emerged as the most influential individual predictors of HEC, and distance to rivers and grassland cover contributed moderate predictive power by themselves (Fig. S6).

### Future conflict projections

**Figure 3:**
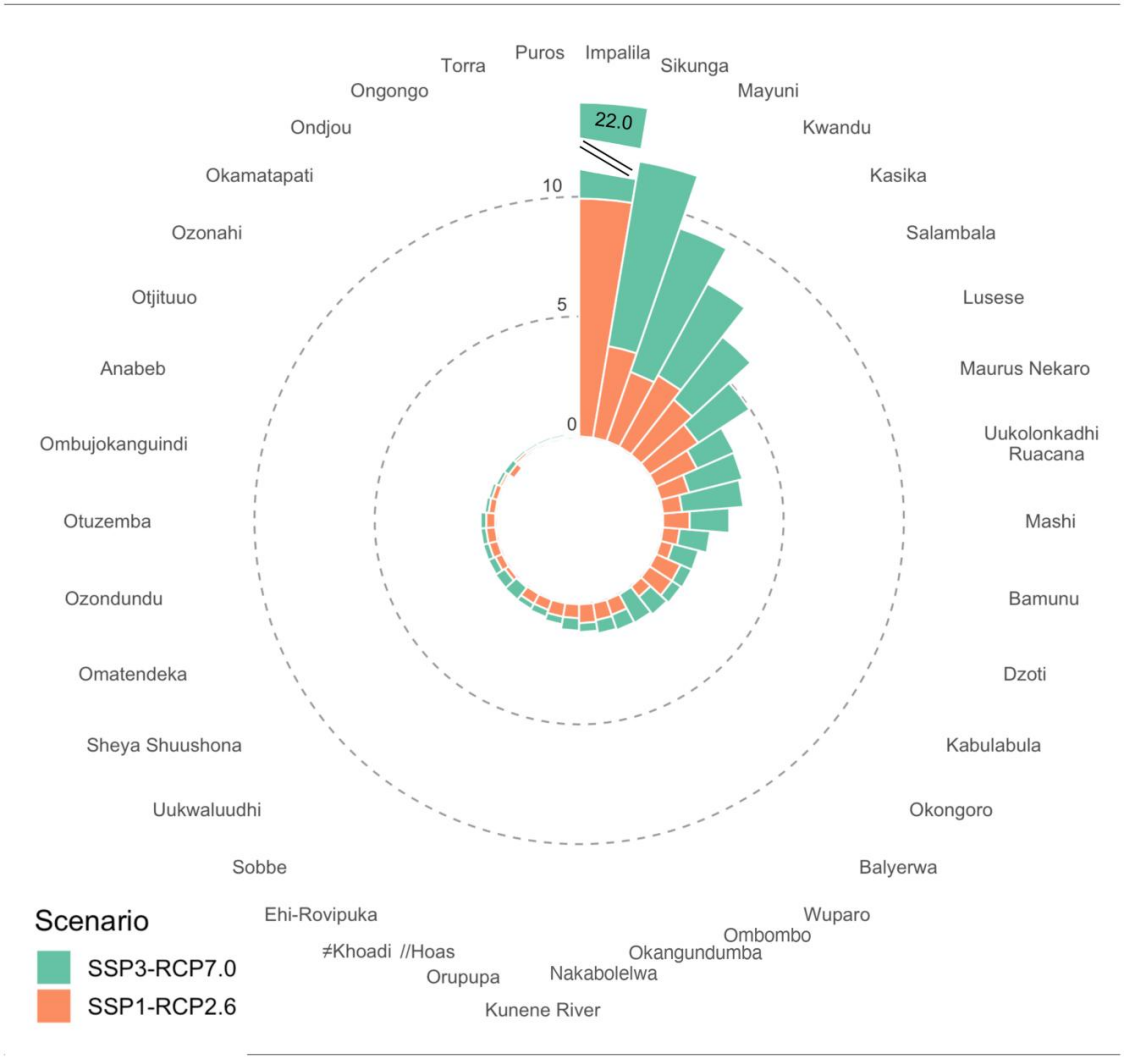
Projections of average grid-level change in HEC events by reporting conservancy, with SSP1-RCP2.6 (“sustainability”) and SSP3-RCP7.0 (“regional rivalry”) projections stacked. Change values for each conservancy are provided in Table S7. See Fig. S12 for the spatial distribution of change values by each reporting conservancy. Figure S19 shows the location of all conservancies that reported HEC across Namibia.

Regression projections represent the expected change in grid-level conflict per year, whereas point process model projections examine changes in the extent of predicted conflict presence. While the scale of change varied by scenario, all scenarios predicted moderate-to-large increases in crop-raiding events (Table S6). SSP3-RCP7.0 (“regional rivalry”) represents a worst-case scenario for HEC, with twice as much wet season crop raiding as seen under the SSP1-RCP2.6 (“sustainability”) scenario (Fig. 3). While SSP5-RCP8.5 (“fossil-fueled development”) saw dramatic climate impacts, it projects less land conversion and moderate population growth for Namibia and resultantly, had relatively lower rates of HEC (Table S6). Our models, therefore, predict the worst HEC increases under more aggressive future expansion of human population and land uses, rather than under the most severe climate disruption.

**Figure 4:**
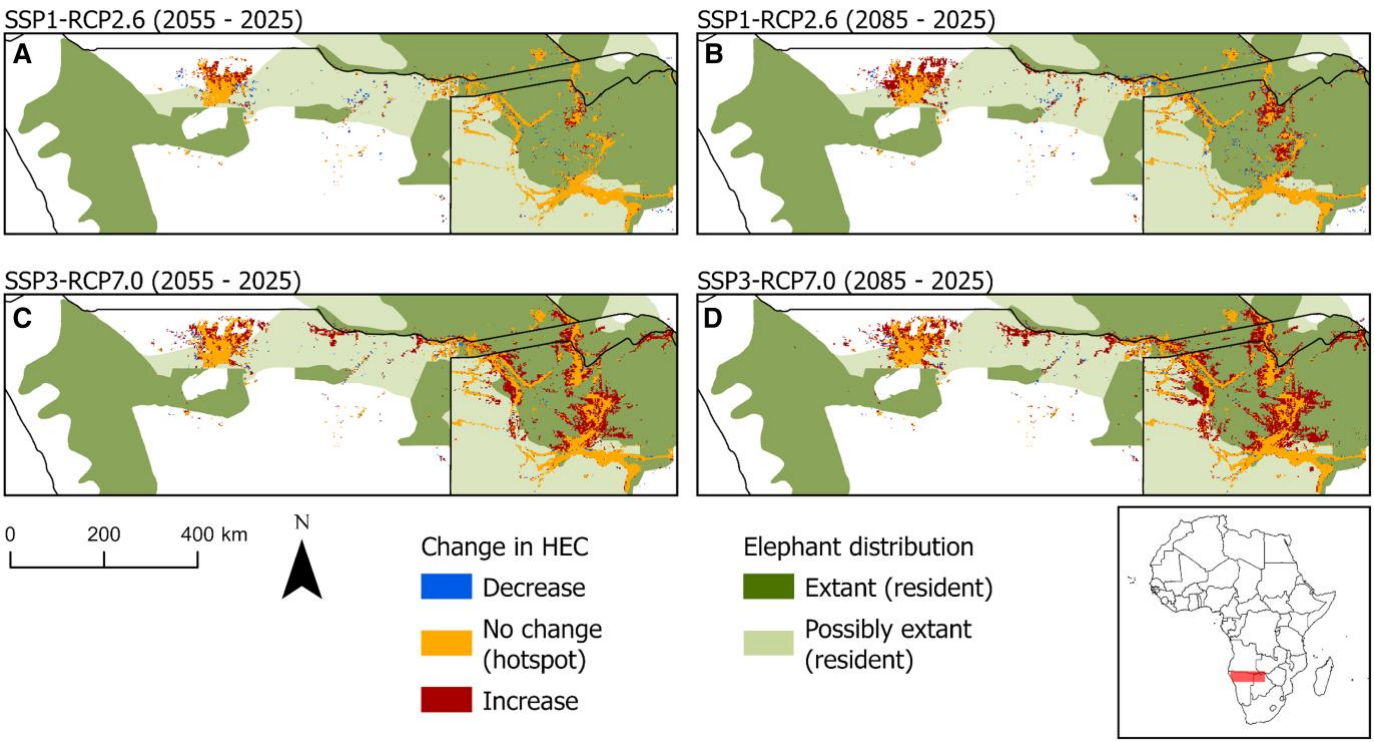
Change in the distribution of crop-raiding HEC during the wet season from 2025 to 2055 and from 2025 to 2085 under two future scenarios: SSP1-RCP2.6 (“sustainability”) and SSP3-RCP7.0 (“regional rivalry”). Continuous HEC predictions were converted to binary outcomes (presence or absence of HEC) using the threshold (0.72) that maximized Cohen's Kappa statistic for the point process model. African elephant distribution from the IUCN is overlaid to compare model results with extant and possibly extant delineations. All regions not colored in the figure were consistent “coldspots” of HEC (i.e. areas with a HEC probability <0.72 in both periods being compared).

The point process models predict a general increase in the probability of crop-raiding HEC toward the end of the century under all emission scenarios in both wet and dry seasons throughout the study area (Figs. S13 and S14), aligning with regression-based estimates. Conflict levels were lowest under SSP1-RCP2.6 (“sustainability”), and highest under SSP3-RCP7.0 (“regional rivalry”; Fig. 4). Predicted hotspots were similar to the baseline point process model results (Fig. 2), with concentrations north of Etosha National Park and along the southwestern boundary of the Zambezi Region in Namibia, and along the periphery of the Okavango Delta and the eastern edge of Moremi Wildlife Reserve in Botswana. Comparisons of areas seeing the most increase in HEC between point process model and regression outputs revealed substantial discrepancies (Figs. S15 and S16), likely due to the regressions’ spatial fixed effects, which limited projections to within-group deviations and increased sensitivity to population changes. In contrast, point process models incorporated spatial patterns across the full study area, making it more responsive to land-cover changes.

## Discussion

We predict HEC to rise in both frequency and spatial extent under all future scenarios. Both the regression and machine learning approaches consistently project the largest increases under SSP3-RCP7.0, with SSP1-RCP2.6 yielding the lowest conflict levels. When compared with baseline data, these increases represent a trend of increasing overlap and discord between elephants and human populations. Our analysis found that growth of human populations and land uses are the primary drivers of future HEC changes, with climate-driven water deficits potentially influencing the movement of elephants out of core areas and into nearby agricultural areas.

Comparing the outputs of our two modeling approaches highlights their complementarity: regression models identified human-driven changes through within-group variation, while point process models captured fine-scale spatial gradients and unimodal relationships. Furthermore, the random effects regressions (Table S4) and point process models (Fig. S6) similarly find that human landscape features like roads and wildlife fences predict HEC risk. When considered together, these models suggest that HEC emerges from both dynamic anthropogenic pressures (population growth and land conversion) and relatively static landscape conditions (proximity to fences, roads, and rivers) that create persistent interfaces for human–elephant interaction. The convergence of two methodologically distinct approaches on the same core conclusion, that anthropogenic landscape features and expansion are the primary drivers of HEC, strengthens confidence in this finding. Integrating these complementary perspectives provides a broader understanding of where and under what conditions HEC will intensify.

Elephant populations across our study area have been recovering from decades of poaching but are increasingly limited to a narrower range of available habitat, potentially increasing resource competition and spillovers of elephants into agricultural areas (4). For example, regions across northwest Botswana where subsistence farming communities share and compete for resources with growing elephant populations are known HEC hotspots and have seen significant increases in HEC over the past two decades (52). Our analysis finds that increasing human land use will continue to place pressure on elephants and is likely to drive increased conflict between humans and elephants, emphasizing the risks of elephant range overlap with cropland areas (15). Research at the scale of common HEC mitigation techniques like “elephant-sensitive” land-use planning, local scout-based monitoring systems, and elephant deterrents can further support local adaptation to HEC (11, 16, 53).

This study links future climate and development scenarios to projected increases in HEC in Southern Africa. Previous research has highlighted the expansion of human land use into elephant ranges (15), a trend that is mirrored in overall human and wildlife populations, which are expected to increase in overlap across 56% of global terrestrial land area through 2070 (2). Our finding of a clear causal link between recent human footprint expansion and rising conflict underscores the concerning predictions of globally increasing overlap between humans and wildlife species. Research suggests that human-dominated landscapes can provide functional connectivity between less disturbed habitats despite having unsustainably high mortality rates for wildlife (54); however, species persistence does not solve the underlying harms that conflict causes to human lives and livelihoods (9) 55). These livelihood impacts, along with support for long-term conservation measures needed for the preservation of species and natural systems, are of utmost concern for regions experiencing wildlife-induced conflict globally.

Interpretation of our findings should consider key limitations related to data usage, spatial resolution, and assumptions about future environmental and demographic conditions. Our projections assume a static elephant population distribution; future changes in elephant abundance or movement corridors could independently amplify or dampen conflict patterns. Furthermore, the HEC dataset may underreport true occurrences due to gaps in the community-based reporting system, potentially affecting model accuracy. Because spatially explicit future projections of roads, fences, and other infrastructure were unavailable, these variables were held constant in the scenario projections, and these results should be interpreted as changes in HEC conditional on the current configuration of those landscape features. Additionally, land-cover data used in this analysis were based on generalized plant functional types, limiting our ability to assess crop-specific variation in raiding risk, which has previously been observed in the eastern Okavango Panhandle of Botswana (56). Lastly, climate projections, while downscaled, may not capture localized orographic and hydrological variation that influences conflict dynamics. A more detailed discussion of these and other limitations, including model fitting considerations and population trend assumptions, is provided in Text S3.

## Conclusion

The ongoing protection of the 290,000 elephants in Southern Africa today (3) represents a positive conservation success story, but conflict with human communities puts this success at risk. Our findings reveal that, without proactive intervention, HEC in our study area is projected to rapidly increase in number and extent through the end of the century. This has the potential to further impact human lives and livelihoods and undermine the region's recent progress in wildlife conservation. However, our finding that anthropogenic land use outweighs climate factors empowers local decision-makers to shape HEC outcomes through proactive landscape planning, an urgent need as human–wildlife overlap intensifies globally. By identifying drivers and future HEC pathways, we provide crucial information to target mitigation measures, support coexistence, and protect human livelihoods and at-risk species into the coming decades. More broadly, our integrated causal-inference and machine learning framework provides a transferable approach for forecasting and managing human–wildlife conflict under global change.

## Supplementary Material

pgag205_Supplementary_Data

## Data Availability

Replication code and data for this study are provided at (57) and can be found at doi:10.5281/zenodo.19561229. Analysis was performed in R (version 4.2.1) and the authors specifically use the dismo R package (version 1.3.14) and the ENMeval R package (version 2.0.4) for point process model selection and creation, and the “plm” R package (version 2.6-6) for the fixed-effects linear models.

## References

[1] Abrahms B, et al 2023. Climate change as a global amplifier of human–wildlife conflict. Nat Clim Chang. 13:224–234.

[2] Ma D, et al 2024. Global expansion of human-wildlife overlap in the 21^st^ century. Sci Adv. 10:eadp7706. 39167651 10.1126/sciadv.adp7706 PMC11338222

[3] Huang RM, Maré C, Guldemond RA, Pimm SL, van Aarde RJ. 2024. Protecting and connecting landscapes stabilizes populations of the Endangered savannah elephant. Sci Adv. 10:eadk2896. 10.1126/sciadv.adk2896 38181078 PMC10776014

[4] Stoldt M, Göttert T, Mann C, Zeller U. 2020. Transfrontier conservation areas and human-wildlife conflict: the case of the Namibian component of the Kavango-Zambezi (KAZA) TFCA. Sci Rep. 10:7964. 10.1038/s41598-020-64537-9 32409783 PMC7224369

[5] Mwangi DK, et al 2016. Socioeconomic and health implications of human wildlife interactions in Nthongoni, Eastern Kenya. Afr J Wildl Res. 46:87–102.

[6] Nsonsi F, Heymans J-C, Diamouangana J, Mavinga FB, Breuer T. 2018. Perceived human–elephant conflict and its impact for elephant conservation in northern Congo. Afr J Ecol. 56:208–215.

[7] Shaffer LJ, Khadka KK, Van Den Hoek J, Naithani KJ. 2019. Human-elephant conflict: a review of current management strategies and future directions. Front Ecol Evol. 6:235. 10.3389/fevo.2018.00235

[8] Abrahms B . 2021. Human-wildlife conflict under climate change. Science. 373:484–485. 34326219 10.1126/science.abj4216

[9] Drake MD, et al 2021. Costs of elephant crop depredation exceed the benefits of trophy hunting in a community-based conservation area of Namibia. Conserv Sci Pract. 3:e345.

[10] Branco PS, et al 2019. Determinants of elephant foraging behaviour in a coupled human-natural system: is brown the new green? J Anim Ecol. 88:780–792. 30825191 10.1111/1365-2656.12971

[11] Jayakody S, et al 2024. Maxent modeling for predicting the potential distribution of human-elephant conflict risk in Sri Lanka. Appl Geogr. 173:103447. 10.1016/j.apgeog.2024.103447

[12] Munyao M, Siljander M, Johansson T, Makokha G, Pellikka P. 2020. Assessment of human–elephant conflicts in multifunctional landscapes of Taita Taveta County, Kenya. Glob Ecol Conserv. 24:e01382. 10.1016/j.gecco.2020.e01382

[13] Thant ZM, May R, Røskaft E. 2021. Pattern and distribution of human-elephant conflicts in three conflict-prone landscapes in Myanmar. Glob Ecol Conserv. 25:e01411.

[14] Dejene SW, Mpakairi KS, Kanagaraj R, Wato YA, Mengistu S. 2021. Modelling continental range shift of the African elephant (*Loxodonta africana*) under a changing climate and land cover: implications for future conservation of the species. Afr Zool. 56:25–34.

[15] Guarnieri M, et al 2024. Effects of climate, land use, and human population change on human–elephant conflict risk in Africa and Asia. Proc Natl Acad Sci U S A. 121:e2312569121. 38285935 10.1073/pnas.2312569121 PMC10861898

[16] Graham MD, Notter B, Adams WM, Lee PC, Ochieng TN. 2010. Patterns of crop-raiding by elephants, *Loxodonta africana*, in Laikipia, Kenya, and the management of human–elephant conflict. Syst Biodivers. 8:435–445.

[17] Mukeka JM, Ogutu JO, Kanga E, Røskaft E. 2019. Human-wildlife conflicts and their correlates in Narok County, Kenya. Glob Ecol Conserv. 18:e00620. 10.1016/j.gecco.2019.e00620

[18] Naha D, Sathyakumar S, Dash S, Chettri A, Rawat GS. 2019. Assessment and prediction of spatial patterns of human-elephant conflicts in changing land cover scenarios of a human-dominated landscape in North Bengal. PLoS One. 14:e0210580. 10.1371/journal.pone.0210580 30707690 PMC6358066

[19] Rani M, et al 2024. Assessment and prediction of human-elephant conflict hotspots in the human-dominated area of Rajaji-Corbett landscape, Uttarakhand, India. J Nat Conserv. 79:126601. 10.1016/j.jnc.2024.126601

[20] Chen G, Li X, Liu X. 2022. Global land projection based on plant functional types with a 1-km resolution under socio-climatic scenarios. Sci Data. 9:125. 10.1038/s41597-022-01208-6 35354830 PMC8967933

[21] UNEP-WCMC and IUCN. 2024. Protected Planet: The World Database on Protected Areas (WDPA) [Online], Accessed December 2024. Cambridge, UK: UNEP-WCMC and IUCN. 10.34892/6fwd-af11

[22] Huang RM, van Aarde RJ, Pimm SL, Chase MJ, Leggett K. 2022. Mapping potential connections between Southern Africa's elephant populations. PLoS One. 17:e0275791. 36219597 10.1371/journal.pone.0275791 PMC9553058

[23] Craig GC, Gibson DSC, Uiseb KH. 2021. Namibia's elephants—population, distribution and trends. Pachyderm. 62:35–52.

[24] O’Connell-Rodwell CE, Rodwell T, Rice M, Hart LA. 2000. Living with the modern conservation paradigm: can agricultural communities co-exist with elephants? A five-year case study in East Caprivi, Namibia. Biol Conserv. 93:381–391.

[25] Wenborn MJ, et al 2025. Assessment of mitigation of human–elephant conflict in the highlands of Northwest Namibia. J Environ Dev. 34:10704965241311353.

[26] Awala SK, Hove K, Wanga MA, Valombola JS, Mwandemele OD. 2019. Rainfall trend and variability in semi-arid northern Namibia: implications for smallholder agricultural production. Welwitschia Int J Agric Sci. 1:1–25.

[27] Jones B . The evolution of Namibia's communal conservancies. In: Nelson F, editor. Community rights, conservation and contested land: the politics of natural resource governance in Africa. Routledge, 2010. p. 106–120.

[28] Namibian Association of CBNRM Support Organisations (NACSO) . 2025. Communal Conservancies. NACSO. Accessed April 25, 2025. https://nacso.org.na/communal-conservancies/

[29] MEFT & NACSO . 2022. The state of community conservation in Namibia (Annual Report 2022). MEFT/NACSO, Windhoek. Accessed October 12, 2024. https://conservationnamibia.com/other/meft-nacso-state-of-community-conservation-namibia-2022.pdf

[30] Loarie SR, van Aarde RJ, Pimm SL. 2009. Elephant seasonal vegetation preferences across dry and wet savannas. Biol Conserv. 142:3099–3107.

[31] Haklay M, Weber P. 2008. Openstreetmap: user-generated street maps. IEEE Pervasive Comput. 7:12–18.

[32] Linard C, Gilbert M, Snow RW, Noor AM, Tatem AJ. 2012. Population distribution, settlement patterns and accessibility across Africa in 2010. PLoS One. 7:e31743. 22363717 10.1371/journal.pone.0031743 PMC3283664

[33] Bondarenko M . 2020. Individual Countries 1 km Population Density (2000–2020) [Dataset]. University of Southampton. 10.5258/SOTON/WP00674

[34] Harper KL, et al 2023. ESA Land Cover Climate Change Initiative (Land_Cover_cci): Global Plant Functional Types (PFT) Dataset, v2.0.8 [Application/xml]. NERC EDS Centre for Environmental Data Analysis. 10.5285/26A0F46C95EE4C29B5C650B129AAB788

[35] Didan K . 2021. MODIS/Terra vegetation indices 16-Day L3 global 250 m SIN Grid V061. NASA EOSDIS land processes distributed active archive center [Dataset]. 10.5067/MODIS/MOD13Q1.061. Date accessed 9 November 2023.

[36] Tsalyuk M, Kilian W, Reineking B, Getz WM. 2019. Temporal variation in resource selection of African elephants follows long-term variability in resource availability. Ecol Monogr. 89:e01348.

[37] Lehner B, Grill G. 2013. Global river hydrography and network routing: baseline data and new approaches to study the world's large river systems: global river hydrography and network routing. Hydrol Process. 27:2171–2186.

[38] Smith RJ, Kasiki SM. 2000. A spatial analysis of human–elephant conflict in the Tsavo ecosystem, Kenya. Report to the African Elephant Specialist Group, Human–Elephant Conflict Task Force, IUCN. Gland, Switzerland: IUCN/SSC African Elephant Specialist Group; January 2000. 82 p.

[39] US Geological Survey . 2010. Global Multi-resolution Terrain Elevation [Dataset].

[40] Karger DN, et al 2017. Climatologies at high resolution for the earth's land surface areas. Sci Data. 4:170122. 10.1038/sdata.2017.122 28872642 PMC5584396

[41] Muñoz-Sabater J, et al 2021. ERA5-Land: a state-of-the-art global reanalysis dataset for land applications. Earth Syst Sci Data. 13:4349–4383.

[42] Araujo DS, et al 2025. Global future drought layers based on downscaled CMIP6 models and multiple socioeconomic pathways. Sci Data. 12:295. 39971936 10.1038/s41597-025-04612-w PMC11840089

[43] Li M, et al 2022. Spatiotemporal dynamics of global population and heat exposure (2020–2100): based on improved SSP-consistent population projections. Environ Res Lett. 17:094007. 10.1088/1748-9326/ac8755

[44] Greene WH . 2008. The econometric approach to efficiency analysis. Meas Prod Effic Prod Growth. 1:92–250.

[45] Cameron AC, Gelbach JB, Miller DL. 2011. Robust inference with multiway clustering. J Bus Econ Stat. 29:238–249.

[46] Phillips SJ, Anderson RP, Dudík M, Schapire RE, Blair ME. 2017. Opening the black box: an open-source release of Maxent. Ecography. 40:887–893. 10.1111/ecog.03049

[47] Merow C, Smith MJ, Silander JA. 2013. A practical guide to MaxEnt for modeling species’ distributions: what it does, and why inputs and settings matter. Ecography. 36:1058–1069. 10.1111/j.1600-0587.2013.07872.x

[48] Nettleton D . Chapter 6—selection of variables and factor derivation. In: Nettleton D, editor. Commercial data mining. Morgan Kaufmann, 2014. p. 79–104.

[49] Merow C, et al 2014. What do we gain from simplicity versus complexity in species distribution models? Ecography. 37:1267–1281.

[50] Fox J, et al 2012. Package ‘car’. Vienna R Found Stat Comput. 16:333.

[51] Oehlert GW . 1992. A note on the delta method. Am Stat. 46:27–29.

[52] Buchholtz EK, McDaniels M, McCulloch G, Songhurst A, Stronza A. 2023. A mixed-methods assessment of human-elephant conflict in the Western Okavango Panhandle, Botswana. People Nat. 5:557–571.

[53] Karidozo M, Grange La M, Osborn FV. 2016. Assessment of the human wildlife conflict mitigation measures being implemented by the Kavango-Zambezi Transfrontier Conservation Area (KAZA TFCA) partner countries. Final report to the KAZA TFCA Secretariat (BMZ No.: 2009 66 788 and BMZ No.: 2006 65 646), Kasane, Botswana. September 2016, Ver. 6.5. Connected Conservation, https://www.connectedconservation.com/wp-content/uploads/2024/10/ConnectedConservation-HWC-KAZA-TFCA-2016_old.pdf

[54] Lamb CT, et al 2020. The ecology of human–carnivore coexistence. Proc Natl Acad Sci U S A. 117:17876–17883. 32632004 10.1073/pnas.1922097117 PMC7395549

[55] Braczkowski AR, et al 2023. The unequal burden of human-wildlife conflict. Commun Biol. 6:182. 36823291 10.1038/s42003-023-04493-y PMC9950466

[56] Matsika TA, et al 2023. Crop diversity and susceptibility of crop fields to elephant raids in Eastern Okavango Panhandle, Northern Botswana. Ecol Evol. 13:e9910. 10.1002/ece3.9910 36960238 PMC10030231

[57] Yu C, Patrick E, Pepperdine M. 2026. Data and Replication Code for An expanding human footprint will escalate human-elephant conflict in a Southern African landscape through the end of the century [Dataset]. Zenodo. 10.5281/zenodo.19561229

