# Supplementary material for: An expanding human footprint drives escalating human–elephant conflict across a transboundary African landscape through 2085
Source: PNAS Nexus. 2026 Jul 7;5(7):pgag205. doi: 10.1093/pnasnexus/pgag205 (PMC13339083; doi:10.1093/pnasnexus/pgag205)
Supplement: pgag205_Supplementary_Data [file pgag205_supplementary_data.docx]

**
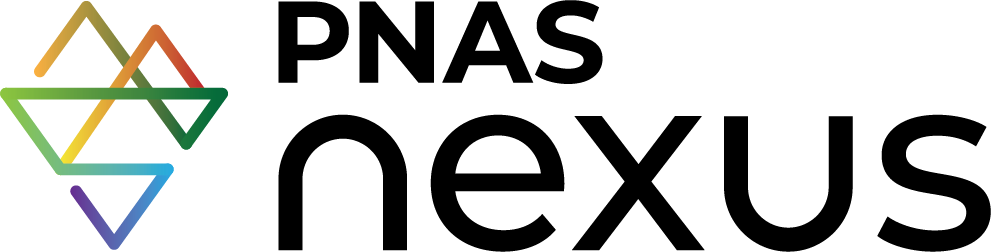
**

**Supplementary Information for**

**An expanding human footprint drives escalating human-elephant conflict across a transboundary African landscape through 2085**

Evan Patrick^1^, Maxwell Pepperdine^1^, Christy Yu^1^, Sophie Pesek^2^, Olivia Somhegyi^3^, Joana M. Krieger^4^, Nickolas McManus^4^, Ezequiel Fabiano^5^, Colgar Sikopo^6^, Patrick R. Roehrdanz^4^, Ashley Larsen^1^

^1^Bren School of Environmental Science & Management, University of California, Santa Barbara, 93106-5131, USA. ^2^Energy and Resources Group, University of California, Berkeley, 345 Giannini Hall, Berkeley, CA 94720, USA. ^3^Conservation Biology Institute, 136 SW Washington Ave., Corvallis, OR 97333, USA. ^4^The Moore Center for Science and Solutions, Conservation International, 2011 Crystal Dr., Arlington, VA, 22202, USA. ^5^Department of Wildlife Management and Tourism Studies, University of Namibia, Katima Mulilo 1096, Namibia. ^6^Ministry of Environment, Forestry and Tourism, Windhoek 13306, Namibia

Corresponding Author: Evan Patrick (epatrick@ucsb.edu)

**Other supplementary materials for this manuscript include the following:**

Text S1 to S3

Figures S1 to S19

Tables S1 to S10

**Introduction**

In this supporting information document, we provide information detailing the regression and maximum entropy analyses performed in this manuscript. This includes further analyses performed, such as random effects and count regression specifications, and information on the point process / maximum entropy variable and model selection. We provide a more detailed description of the limitations of the analysis to supplement the brief limitations discussion provided in the main manuscript text. Additionally, further data is provided on the study system and conservancies analyzed in this study.

# Text S1.

# Alternative Regression Specifications

Crop-raiding events are recorded as non-negative integers that are highly skewed toward zero (Table S1). Treating these data with ordinary least-squares assumes a continuous, homoscedastic, and normally distributed error term. These assumptions may not hold in the context of the HEC event data presented in this analysis. Count regression models were therefore estimated in parallel to the linear fixed-effects analyses to ensure that our inference is robust to the true distributional nature of the response.

Poisson fixed-effects models provide a natural starting point because they are derived from the Poisson process that underlies many arrival-rate phenomena, including rare wildlife-human interactions. However, the variance of crop-raiding counts exceeds the mean (over-dispersion), an issue that inflates Type I error rates in a pure Poisson framework. We therefore also report negative binomial fixed-effects estimates, which introduce an extra dispersion parameter to accommodate unobserved heterogeneity among grid cells and seasons.

Using fixed effects in both Poisson and negative binomial settings retains the key advantage of our linear specification, control for all time-invariant spatial attributes and year-specific shocks, while allowing the conditional mean function to follow the log-link that is appropriate for counts. Comparing coefficient signs, significance, and marginal effects across linear, Poisson, and negative binomial models highlights where conclusions are robust versus sensitive to distributional assumptions. Table S2 and Table S3 present these count-based results, showing a consistent sensitivity to population across seasons, and cropland cover for wet season regressions, aligning with our linear regression results. Built cover was not significant to p<0.05 under either specification but did demonstrate a p<0.10 for the Poisson - wet season model.

Alongside the two-way fixed effects (FE) regressions presented in the main text, we also estimated random effects (RE) panel regressions for both wet and dry season crop-raiding models. While FE models have the advantage of controlling for all time-invariant unobserved heterogeneity within spatial groups, they also difference out the effects of time-invariant predictors (e.g., topography, long-term infrastructure placement) that may be ecologically important for understanding human-elephant conflict (HEC). RE models retain these predictors by allowing the unit-specific intercepts (random effects) to vary across spatial groups while assuming independence between the unobserved effects and the included covariates. This enables the inclusion of both within-group temporal variation and between-group cross-sectional variation, potentially offering a fuller picture of the correlates of HEC risk.

We assessed the appropriateness of RE specifications using the Hausman test, which evaluates whether the RE assumption of independence between unobserved effects and regressors holds. For wet-season crop raiding, the Hausman test rejected RE in favor of FE (p < 0.05), indicating that unobserved heterogeneity is likely correlated with key predictors in that season; these results should therefore be interpreted with caution. For dry-season crop raiding, the test did not reject RE, suggesting that the RE estimator is consistent in this case. Including the RE models alongside FE allows us to explore the role of time-invariant spatial characteristics, such as elevation, slope, and distances to roads, rivers, and fences, while directly comparing how results differ when causal identification relies solely on within-unit temporal changes (FE) versus when both within- and between-unit variation inform the estimates (RE). Results for both wet and dry season RE models are presented in Table S4.

**Text S2.**

# Point Process Model Selection and Evaluation

We used the `ENMeval` R package (version 2.0.4) to generate and evaluate a series of point process (alternatively, Maximum Entropy or MaxEnt) models before selecting the ‘best’ performing model. Two feature class combinations, linear (L) and linear + quadratic (LQ), were used, with regularization multiplier values ranging from 0.5 to 3 in increments of 0.5, to generate a total of 12 models. Using omission rate (OR) and AUC scores as evaluation metrics, the point process model with LQ features and a regularization multiplier of 1 performed the best in the crop raiding model for both the wet (Figure S5) and dry seasons. A single point process model with these parameters was then generated using the `dismo` R package (version 1.3.14), which we used to produce baseline and future predictions of crop raiding HEC events across the study area.

As mentioned, omission rate (OR) and the area under the receiver operating characteristic curve (AUC) were used to assess the models’ predictive performance. OR quantifies the proportion of observed presences that are incorrectly predicted as absences, with lower values indicating better model performance (Phillips et al. 2006; Pearson, 2010). AUC assesses how accurately a model determines presences compared to random prediction (i.e., the model’s ability to distinguish presence locations from background points). Values range from 0 to 1, where values less than 0.5 imply the prediction is worse than random, 0.5 implies random prediction, and 1 implies perfect prediction (Phillips et al. 2006; Pearson, 2010).

**Text S3.**

**Study Limitations**

This study has various limitations related to data usage and modeling efforts. The reporting system used to generate the HEC dataset likely does not capture all HEC occurrences within registered communal conservancies. This may affect fixed-effects and point process model fitting and prediction accuracy, especially if areas with occurrences are measured as having none. However, given the large number of occurrences generated across our study area and period, the omission of some true HEC events is unlikely to significantly impact our results. As with any modeling approach, limitations arise from the parameter selection used to fit and train models. In point process modeling, a key challenge is balancing model complexity to prevent overfitting or underfitting. To mitigate overfitting, we restricted model features to L and LQ. Additionally, we selected the best-performing model based on OR and AUC evaluation metrics. Slight variations in feature class selection or alternative model evaluation criteria (e.g., AIC and BIC) could yield different baseline and future projections when extrapolating point process models to novel combinations of future projections of environmental variables (Merow et al. 2013).

Another limitation concerns land cover data. We relied on a general land cover map with broadly defined plant functional types, using a % cropland indicator to analyze the effects of all crop types on HEC occurrence. This approach does not account for spatial variation in crop-raiding risk across different crop types, which has been observed in the eastern Okavango Panhandle of Botswana (Matsika et al, 2023). More detailed land use datasets that distinguish between crop types could provide finer-scale insights into HEC spatial patterns.

We find that spatial differences in wetness between core areas and grid-level SPEI are predictive of conflict. However, many climate models do not have the spatial specificity to pick up these changes, with downscaling to local conditions often being an imperfect attempt to reflect realized orographic conditions. While the data we use represent some of the best available in downscaled climate information, we are not confident that we have achieved the spatial specificity needed to understand landscape-level differences in weather that could drive conflict. Finally, our analysis relies on the assumption that elephant populations will remain relatively stable over the coming decades. Analyses such as (Huang et al., 2024) have shown that elephant populations across northern Namibia have been growing in recent decades, a trend that could further accelerate opportunities for HEC.


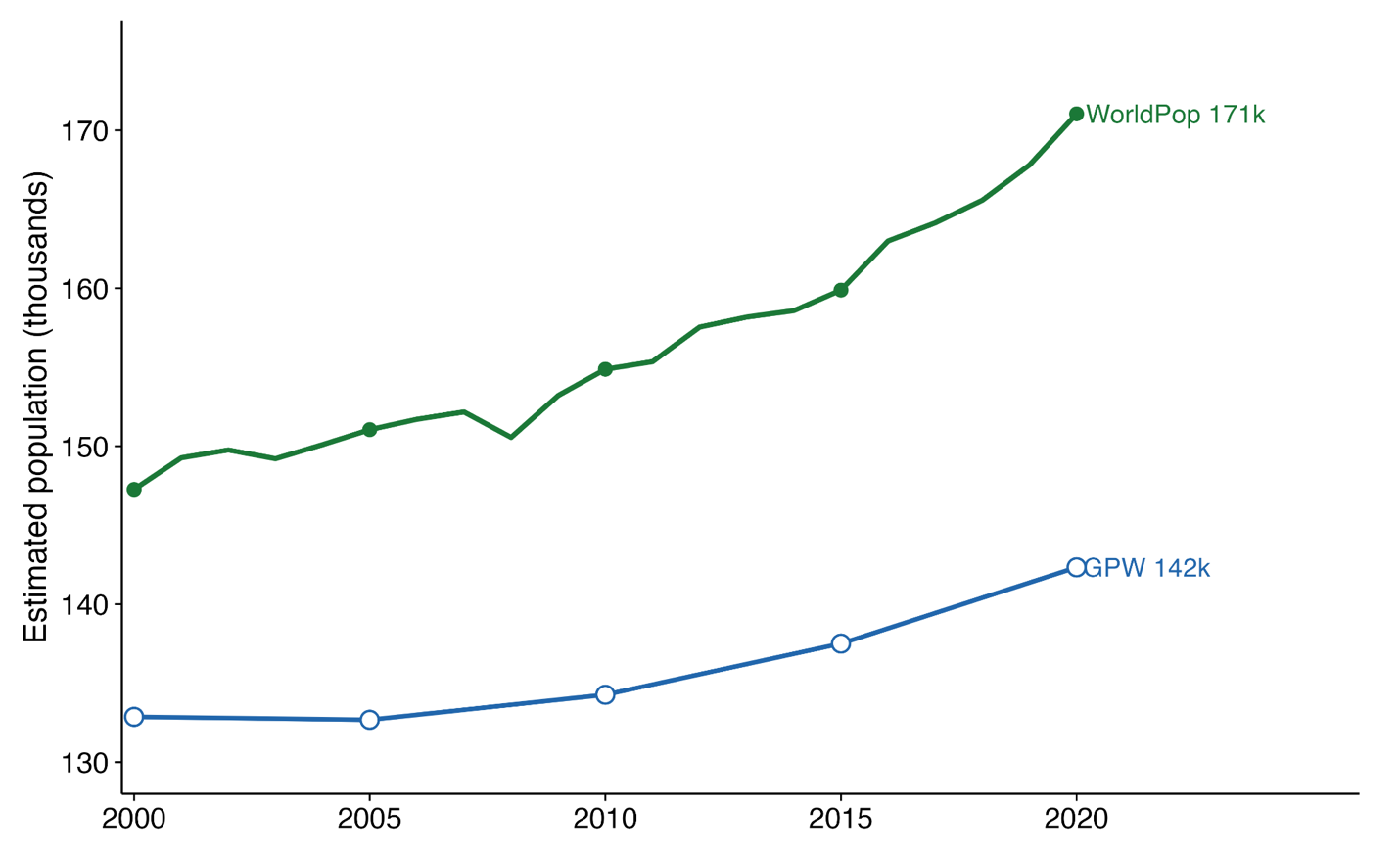


**Fig. S1.** Time-series of population counts for the study area from 2000 to 2020 using two widely-used datasets: WorldPop (Green; Linard et al., 2012; Bondarenko, 2020) and Gridded Population of the World, version 4 (GPW; Blue; CIESIN, 2018). Both datasets show rapid growth (0.34% yearly growth for GPW and 0.75% for WorldPop).


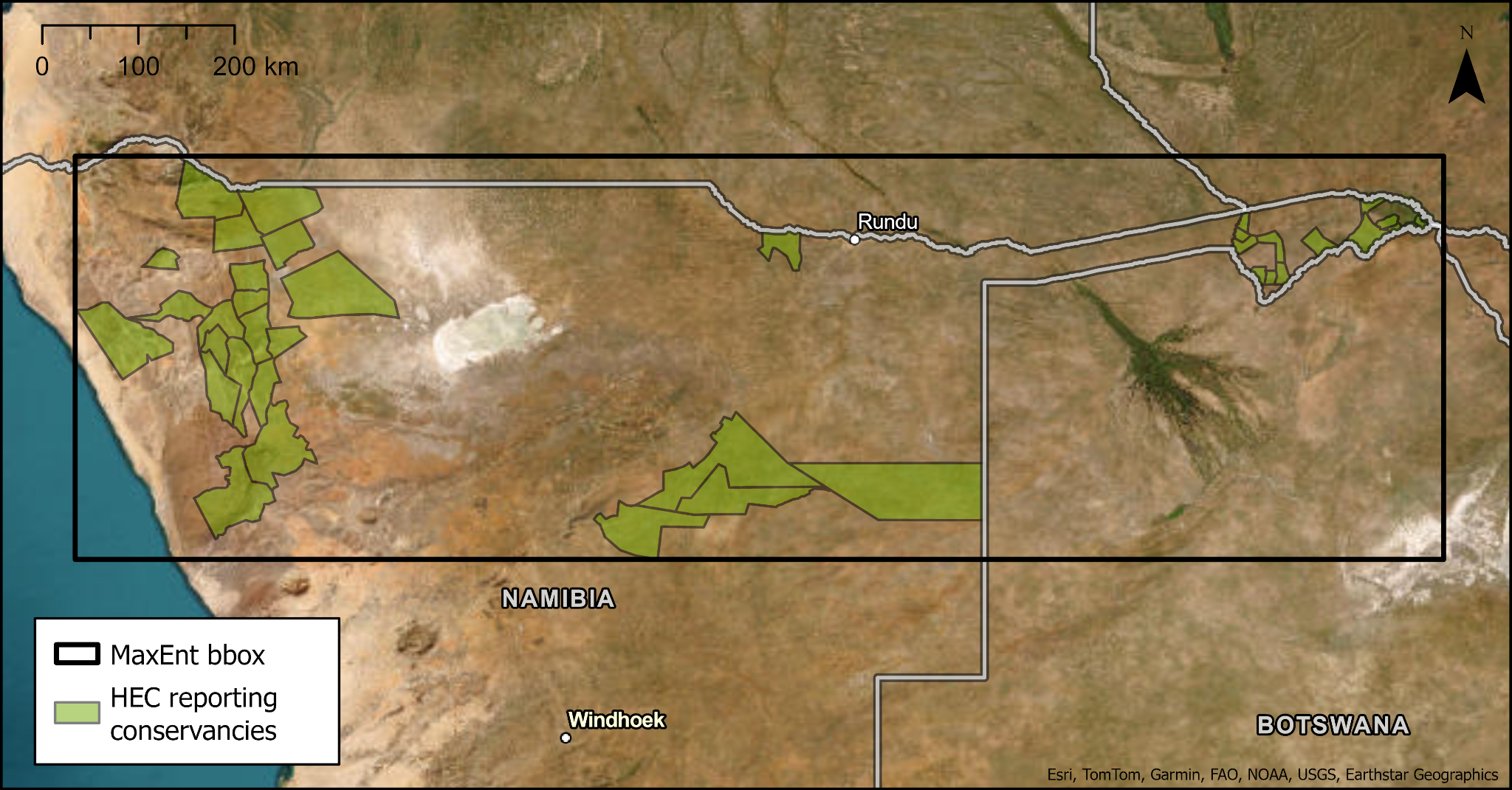
**Fig. S2.** Bounding box (black outline) used to define the extent to which baseline and future point process model predictions of crop raiding HEC probability were projected. Models were trained using HEC occurrence data from within HEC-reporting Namibian communal conservancies (green) and projected across the broader landscape defined by the bounding box (20.900°S to 17.133°S; 12.499°E to 25.283°E). This area encompasses northern Namibia and Botswana, as well as small portions of Angola and Zambia.


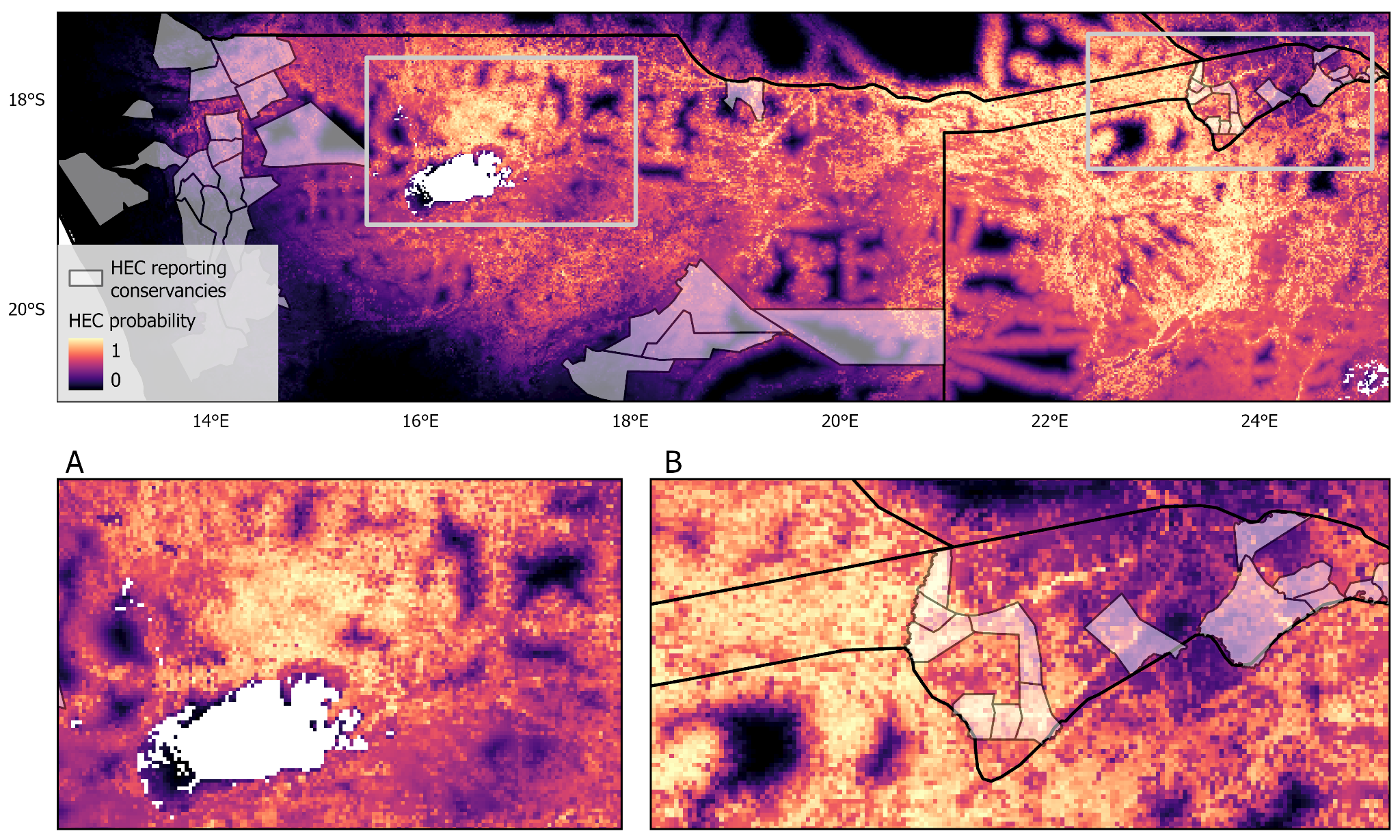


**Fig. S3.** Baseline predictions for crop raiding HEC probability during the 2020 dry season. Two predicted HEC risk hotspots are shown in the inset maps: regions north of the eastern side of Etosha National Park (A) in northwest Namibia and the Zambezi Region (B) in northeast Namibia.


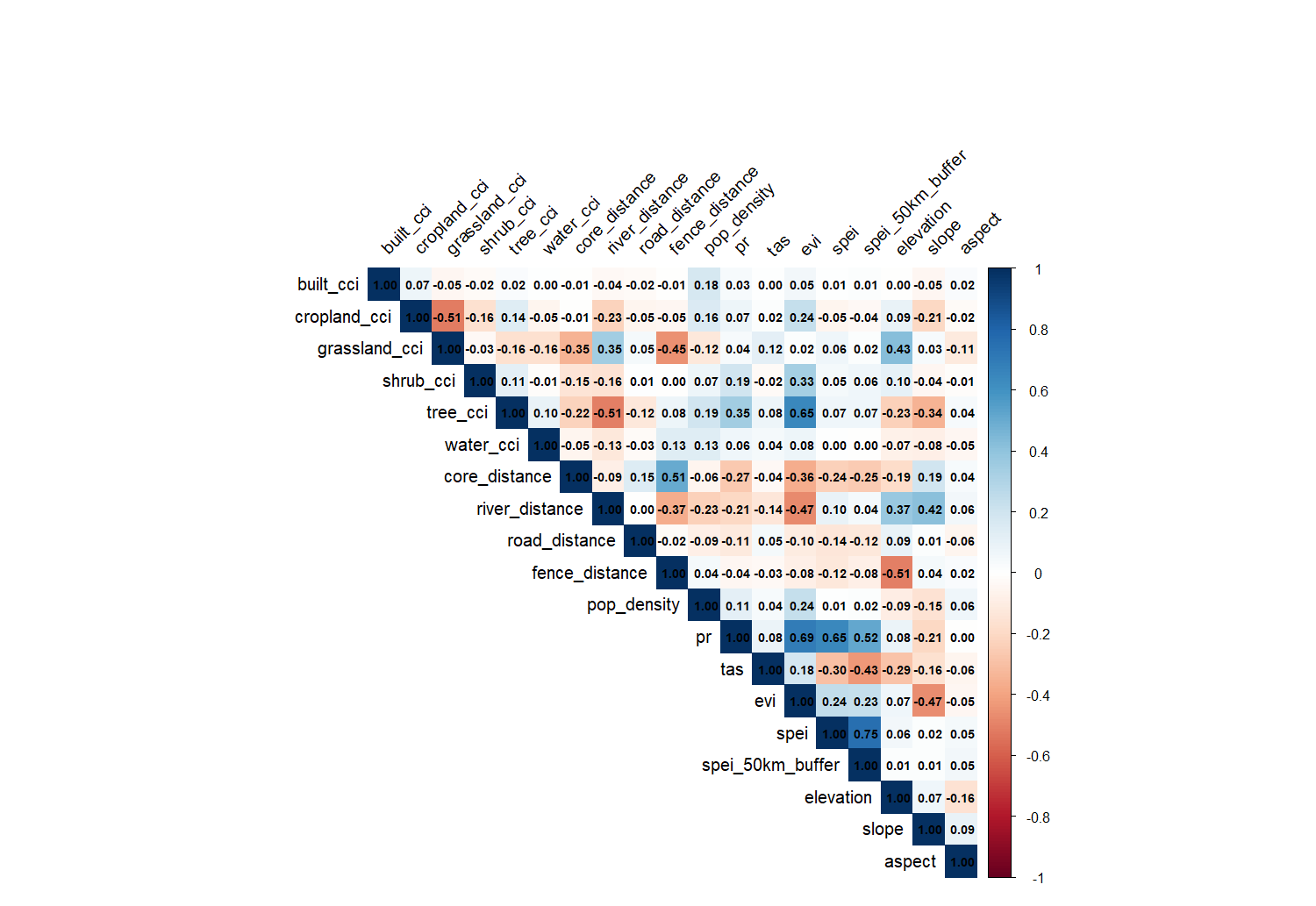


**Fig. S4.** Pearson’s correlation matrix for all environmental predictor variables considered in the point process models.


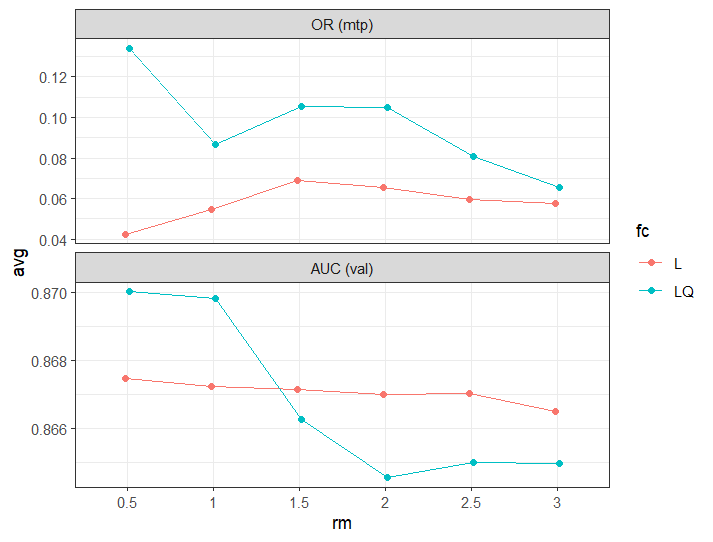


**Fig. S5**. Evaluation metrics used to select the best-performing point process model with crop raiding occurrences during the wet season.


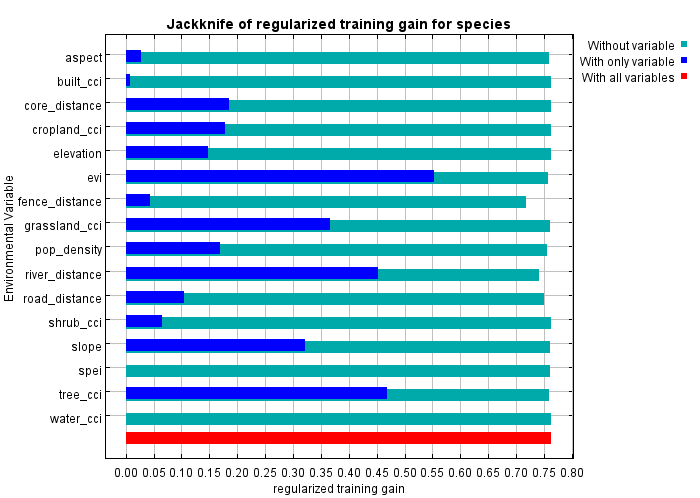


a


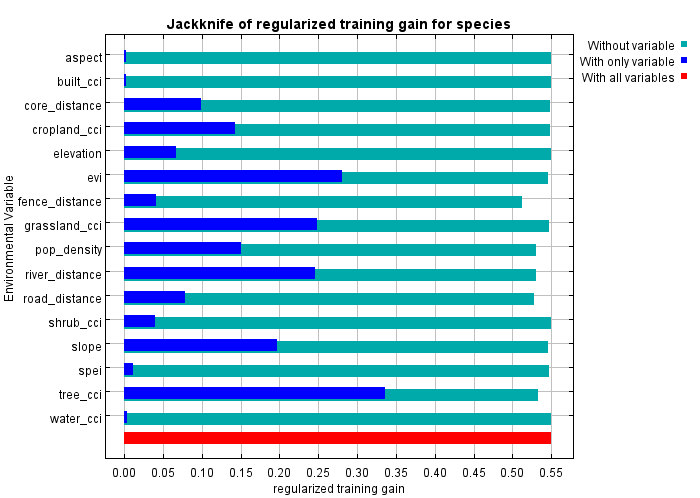


b

**Fig. S6.** Jackknife of regularized training gain for all environmental variables used in the **(a)** crop raiding, wet season point process model and **(b)** crop raiding, dry season point process model. Dark blue shows the regularized training gain with only that variable, teal without that variable, and red with all variables. If a variable alone has high training gain (blue), it’s highly predictive by itself. If the training gain drops significantly when a variable is removed (teal), that variable contributes a lot to the collective model.


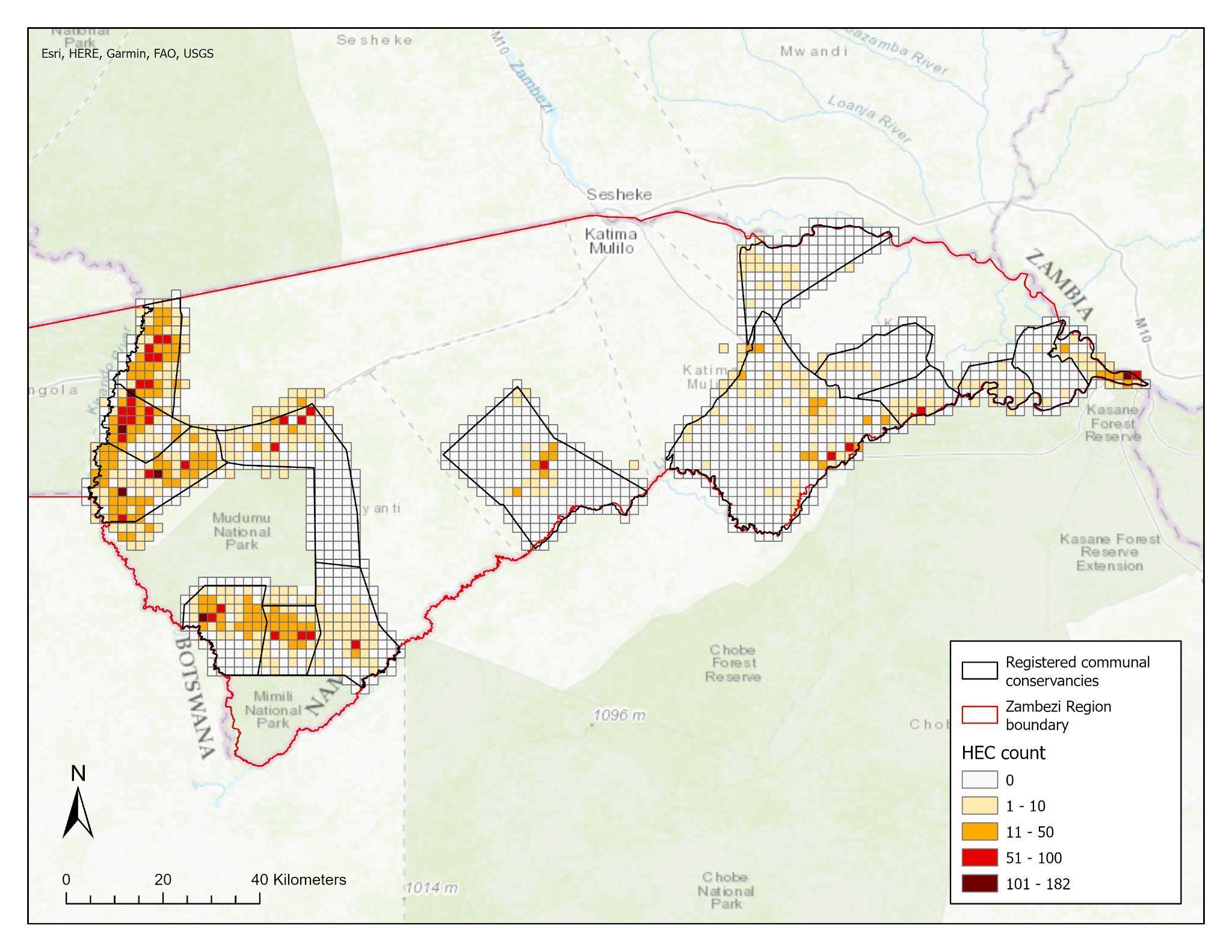


**Fig. S7.** HEC data used in the regression and point process model analyses. Conflict counts are shown for 2004-2020, with higher conflict counts represented by darker shades of red.


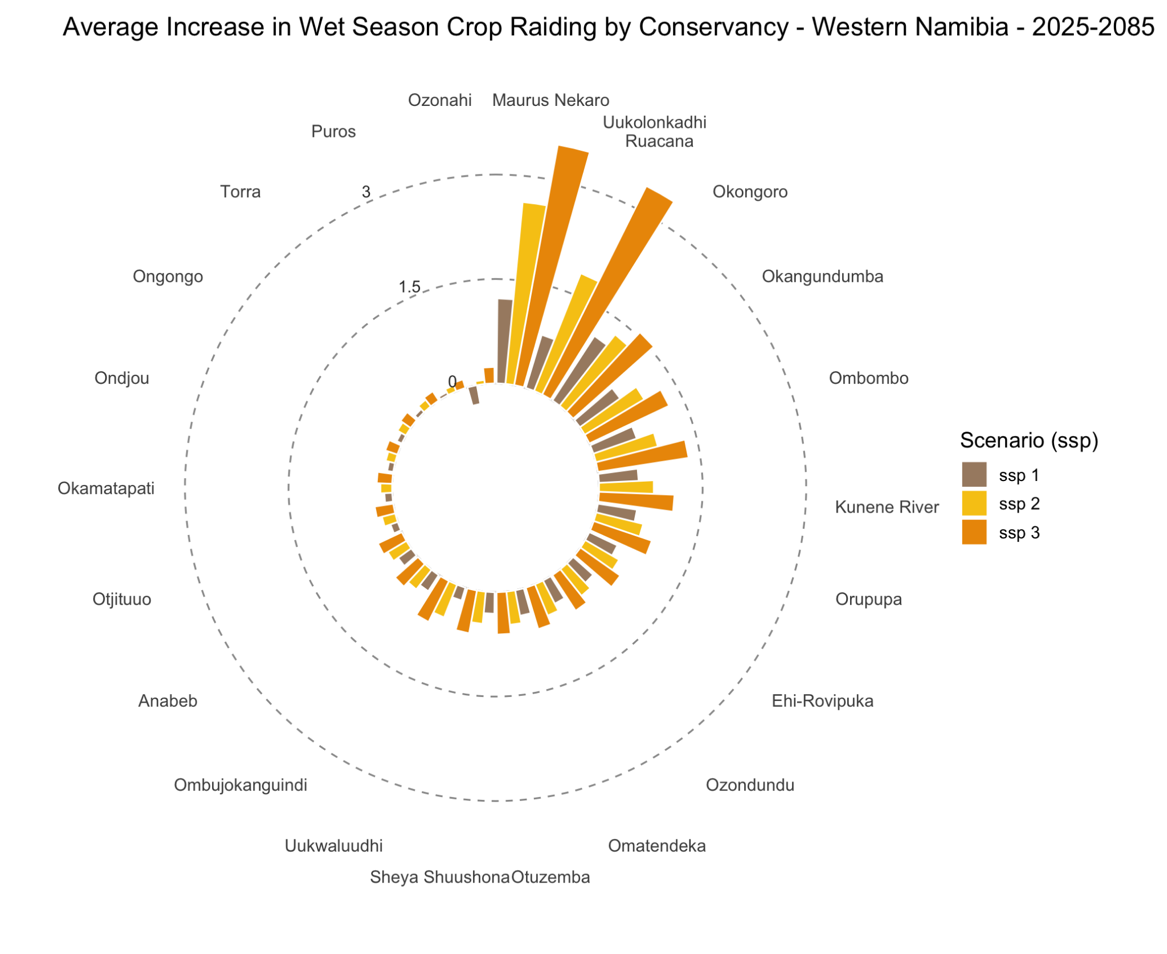


**Fig. S8.** Rose plots of average change in Wet Season Crop Raiding events (per grid cell) from 2025-2085 for Western Namibian conservancies, across SSPs 1, 2, and 3.


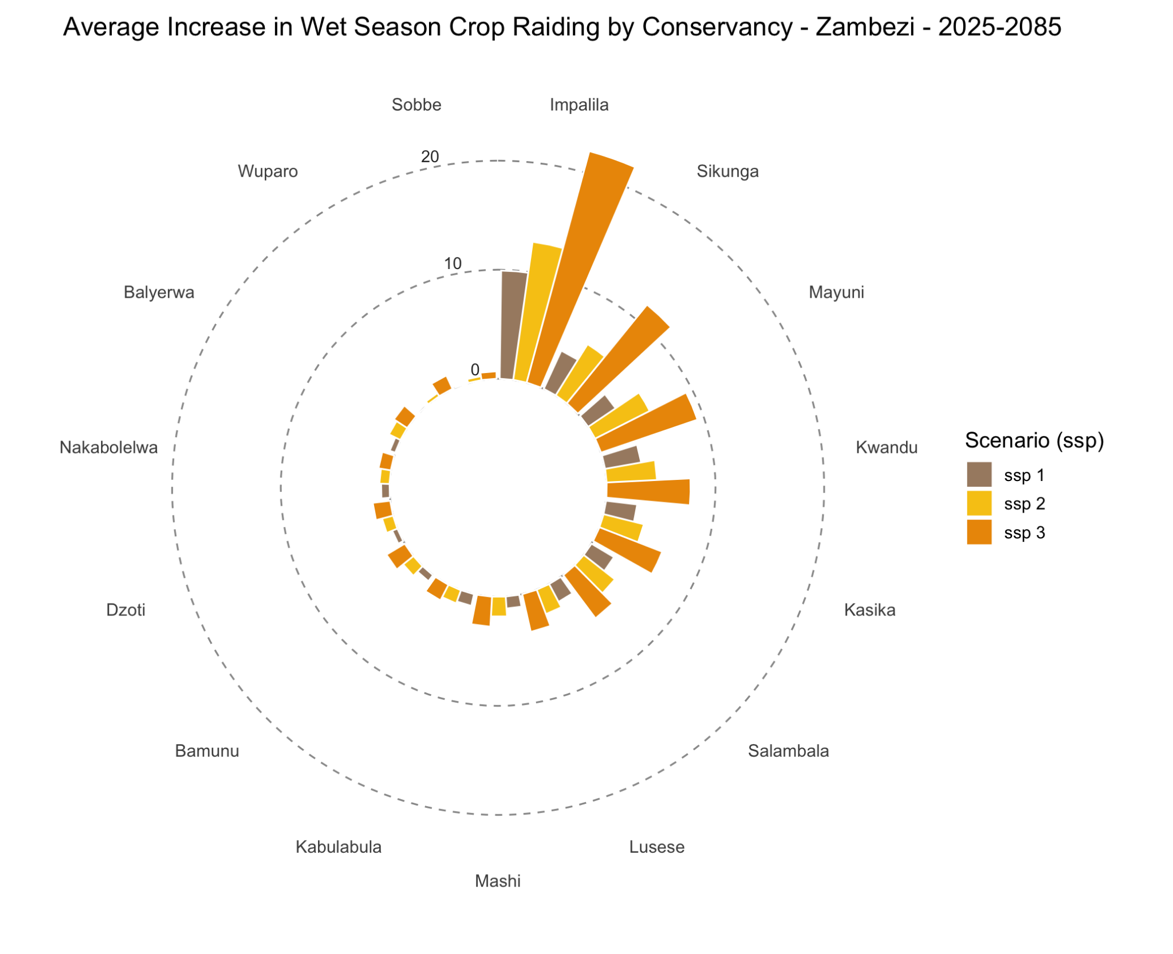


**Fig. S9.** Rose plots of average change in Wet Season Crop Raiding events (per grid cell) from 2025-2085 for conservancies in the Zambezi region, across SSPs 1, 2, and 3.


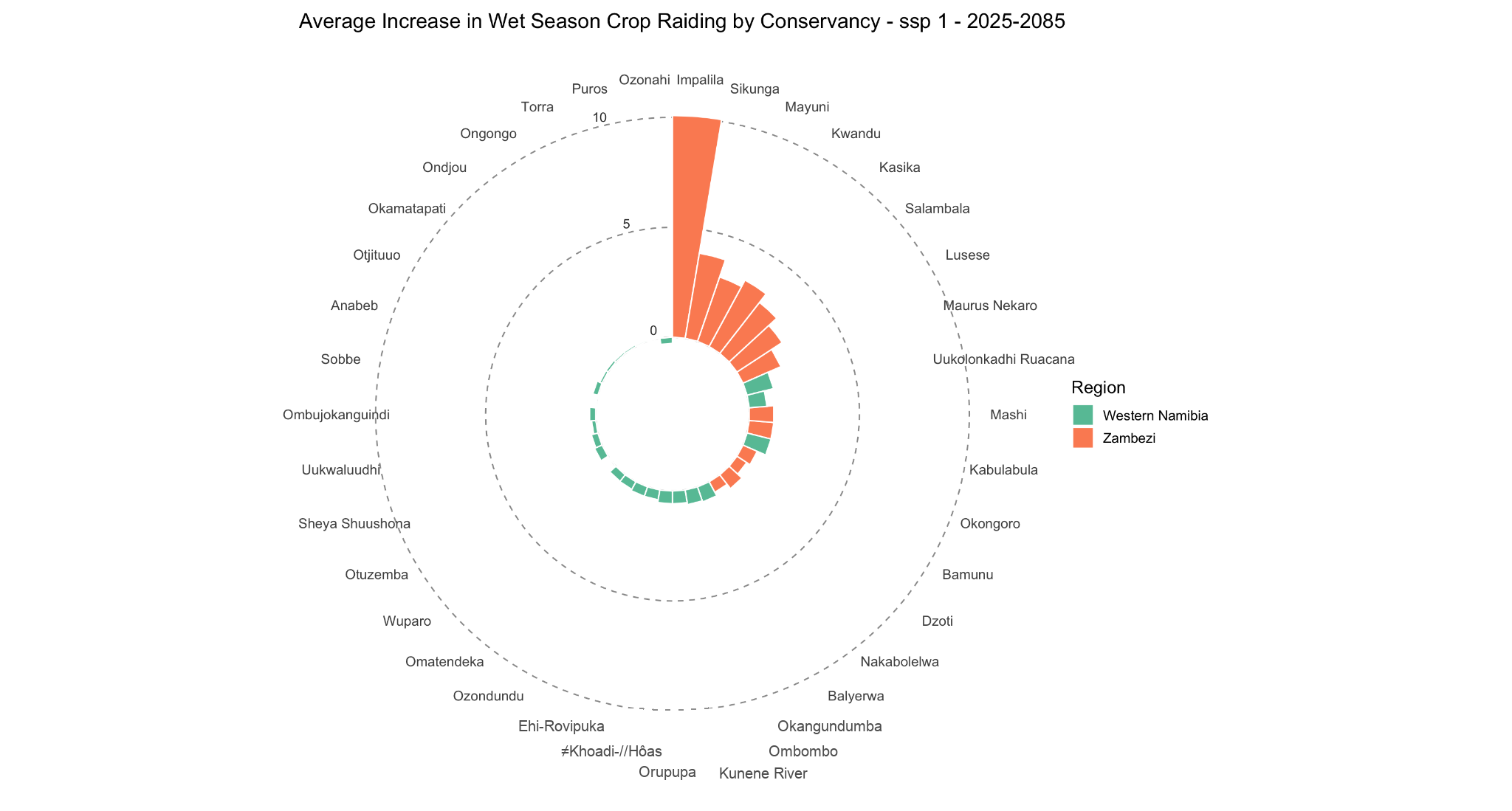
 **Fig. S10**. Rose plots of average grid-cell change in wet season crop raiding events per conservancy from 2025-2085 for SSP 1 RCP 2.6, colored by region.


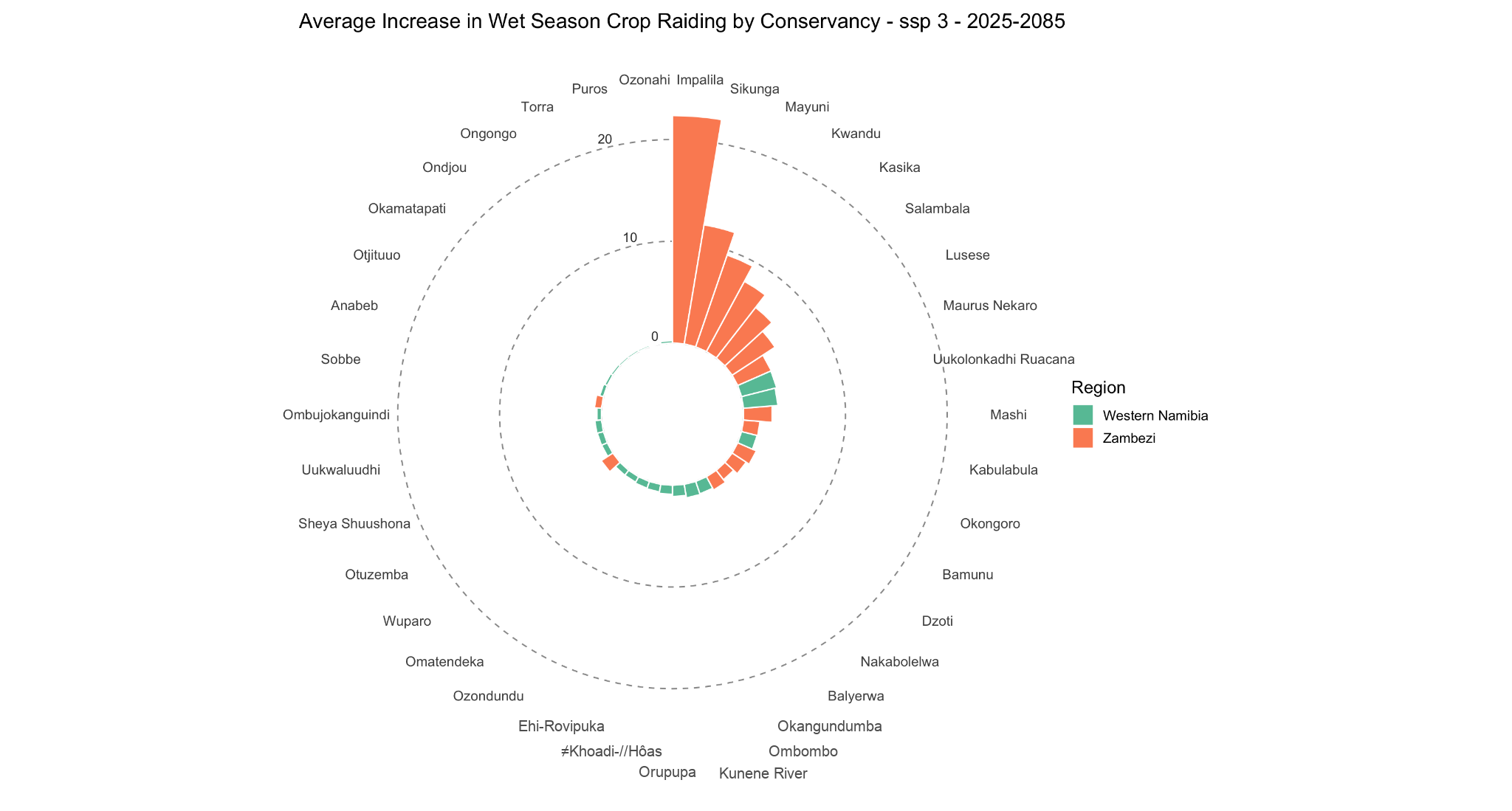


**Fig. S11**. Rose plots of average grid-cell change in wet season crop raiding events per conservancy from 2025-2085 for SSP 3 RCP 7.0, colored by region. Note the difference in scale from Fig. S10.


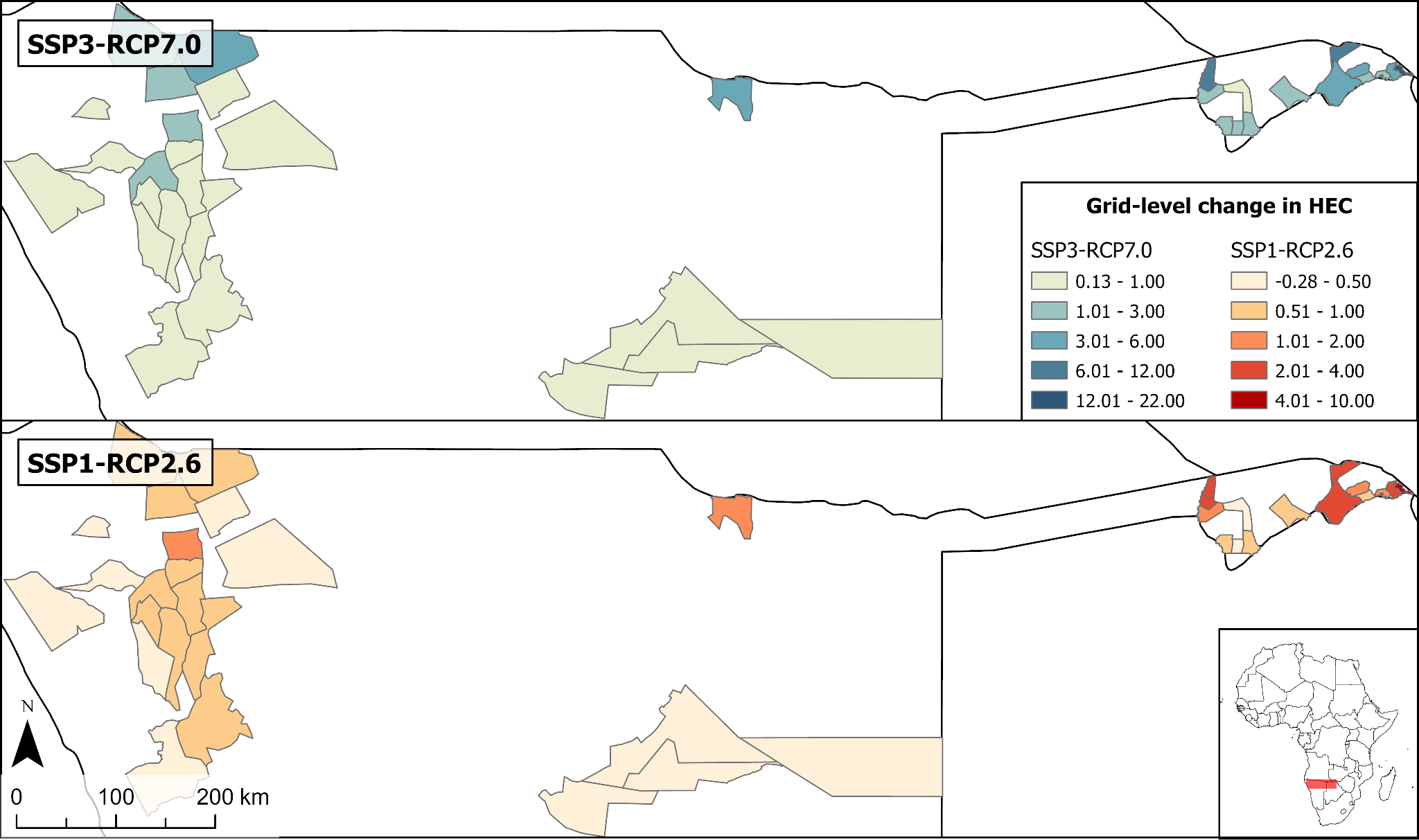
**Fig. S12.** HEC reporting communal conservancies colored by the grid-level change in crop raiding HEC estimates during the wet season for SSP3-RCP7.0 (top) and SSP1-RCP2.6 (bottom). Darker colors represent a greater projected increase in conflict. These are the same values provided in Table S7.


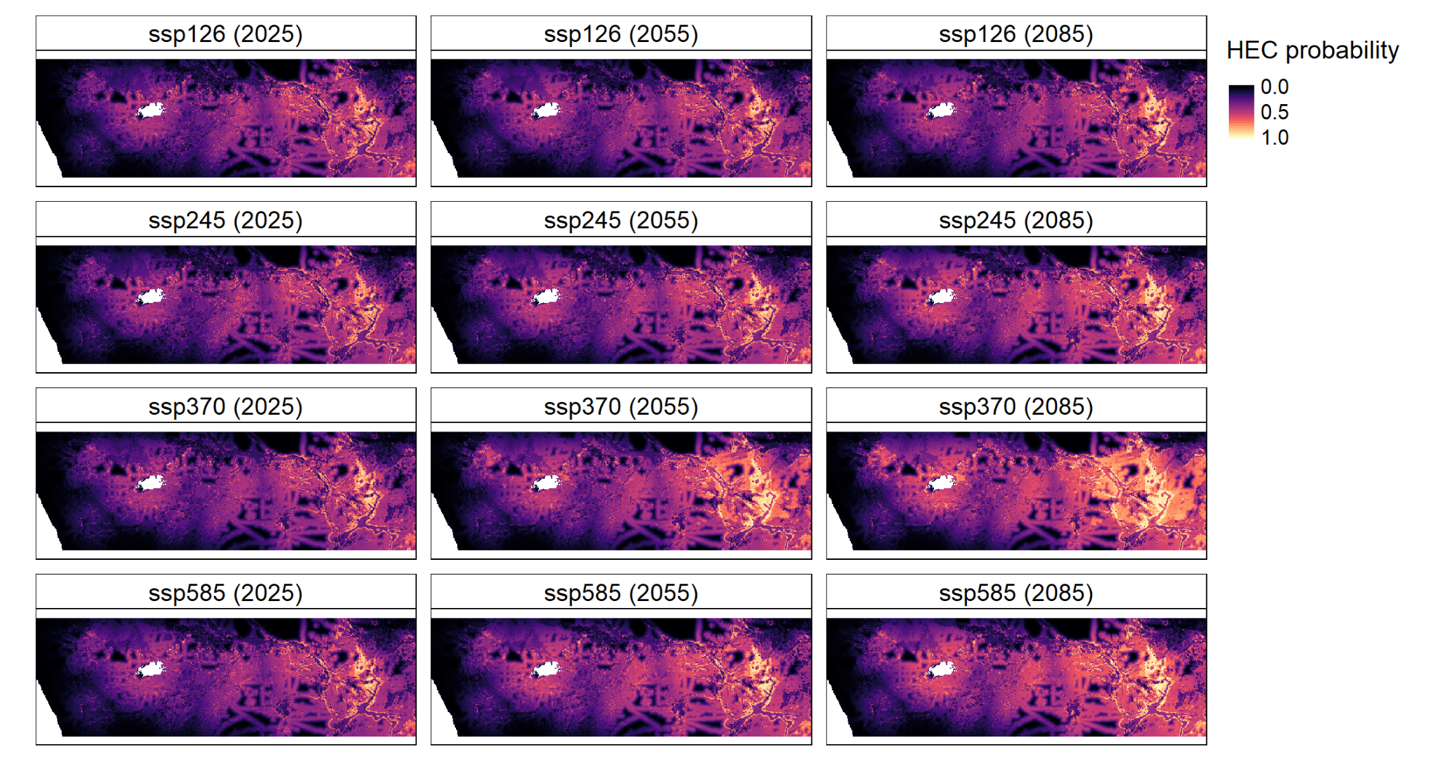


**Fig. S13.** Future HEC projections generated from the point process model for crop raiding conflict event probability during the dry season under four emission scenarios: SSP1-RCP2.6 (Sustainability), SSP2-RCP4.5 (Middle of the Road), SSP3-RCP7.0 (Regional Rivalry), and SSP5-RCP8.5 (Fossil-Fueled Development).


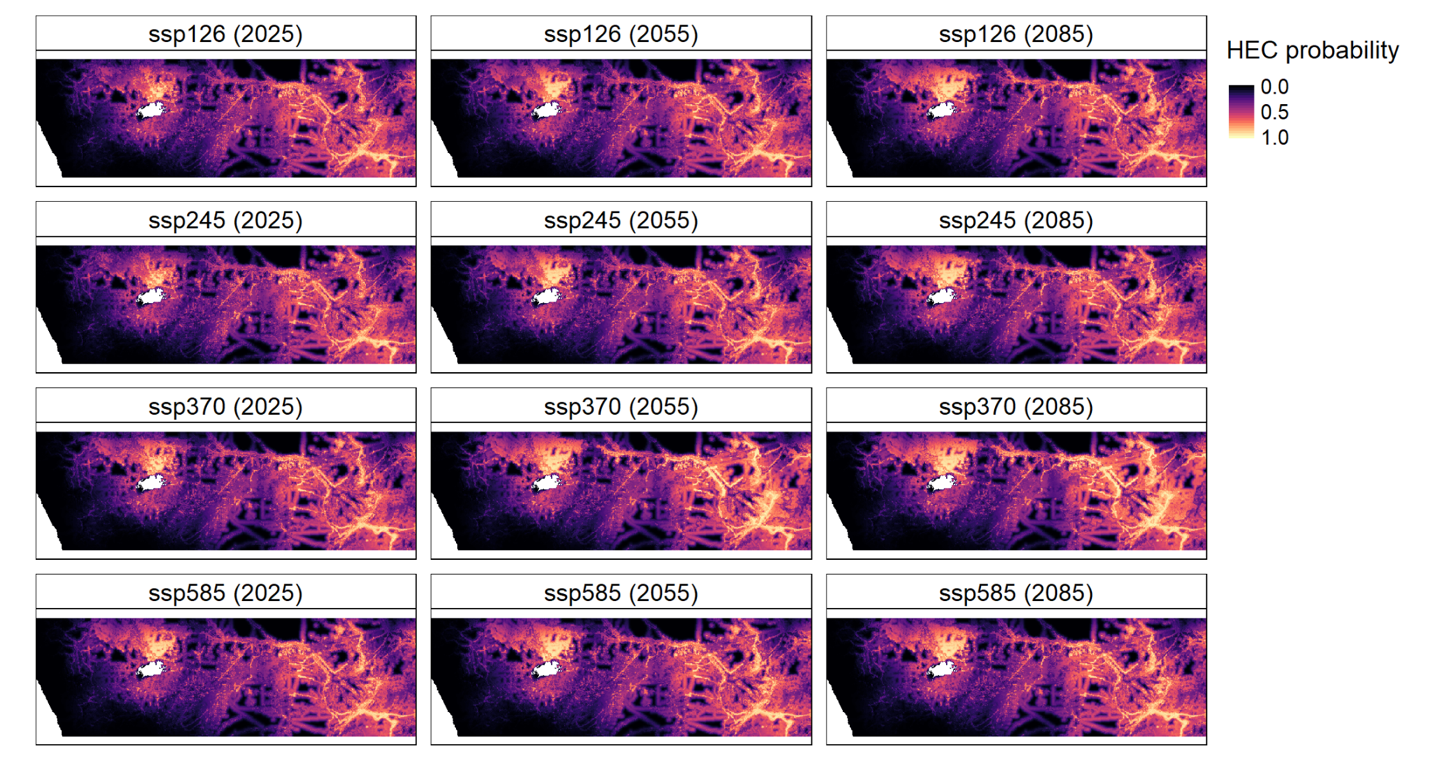


**Fig. S14.** Future HEC projections generated from the point process model for crop raiding conflict event probability during the wet season under four emission scenarios: SSP1-RCP2.6 (Sustainability), SSP2-RCP4.5 (Middle of the Road), SSP3-RCP7.0 (Regional Rivalry), and SSP5-RCP8.5 (Fossil-Fueled Development).


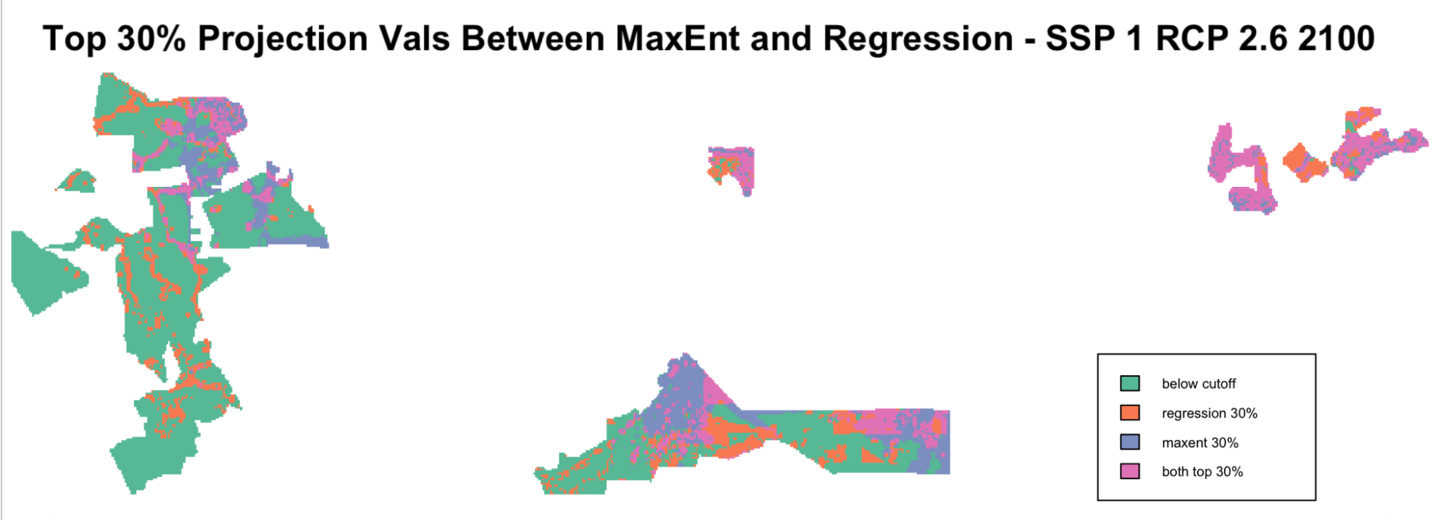


**Fig. S15.** Comparison of the top 30% of projected change pixels between 2025 and 2085 under both the regression and point process models for SSP1-RCP2.6. While there is some consistency between the models, regression outputs showed more linear features in Western Namibia.


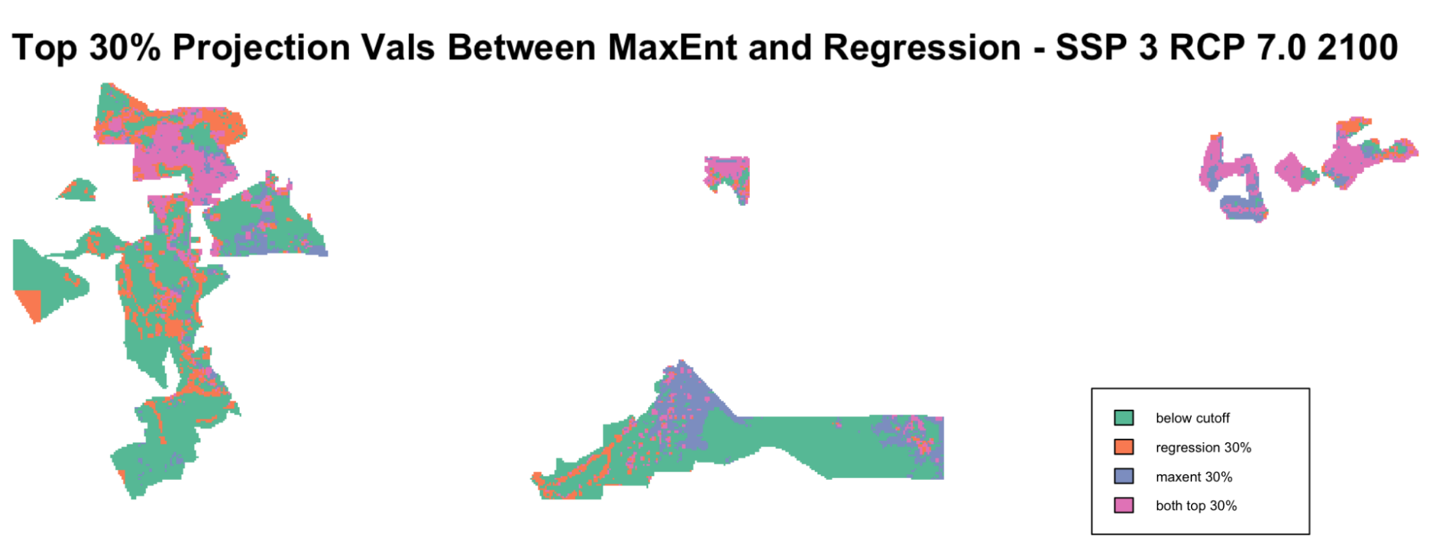
**Fig. S16.** Comparison of the top 30% of projected change pixels between 2025 and 2085 under both the regression and point process models for SSP3-RCP7.0. We see a larger overlap in Northwestern Namibia, as population and crop cover are expected to significantly expand there under this model.


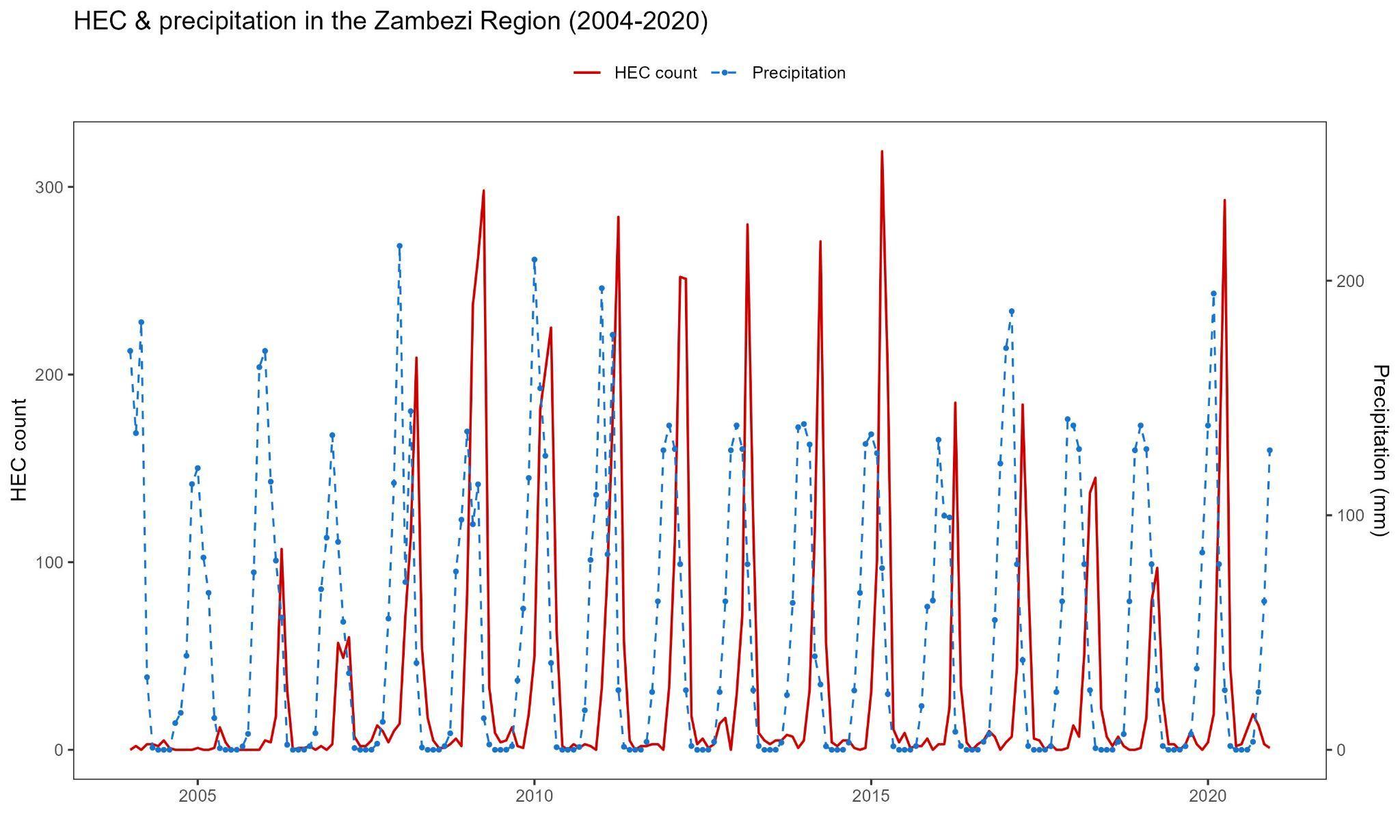
 **Fig. S17.** HEC and mean monthly precipitation (mm) in the Zambezi Region between 2004 and 2020. HEC counts are aggregated monthly across the 15 registered communal conservancies in the Zambezi region, with mean monthly precipitation values shown by the blue dots. Precipitation data was sourced from WorldClim 2.1. Spikes in conflict following the wet season were smaller between 2004 and 2008 because not all registered communal conservancies were reporting conflict during this time, as shown in Table S9.


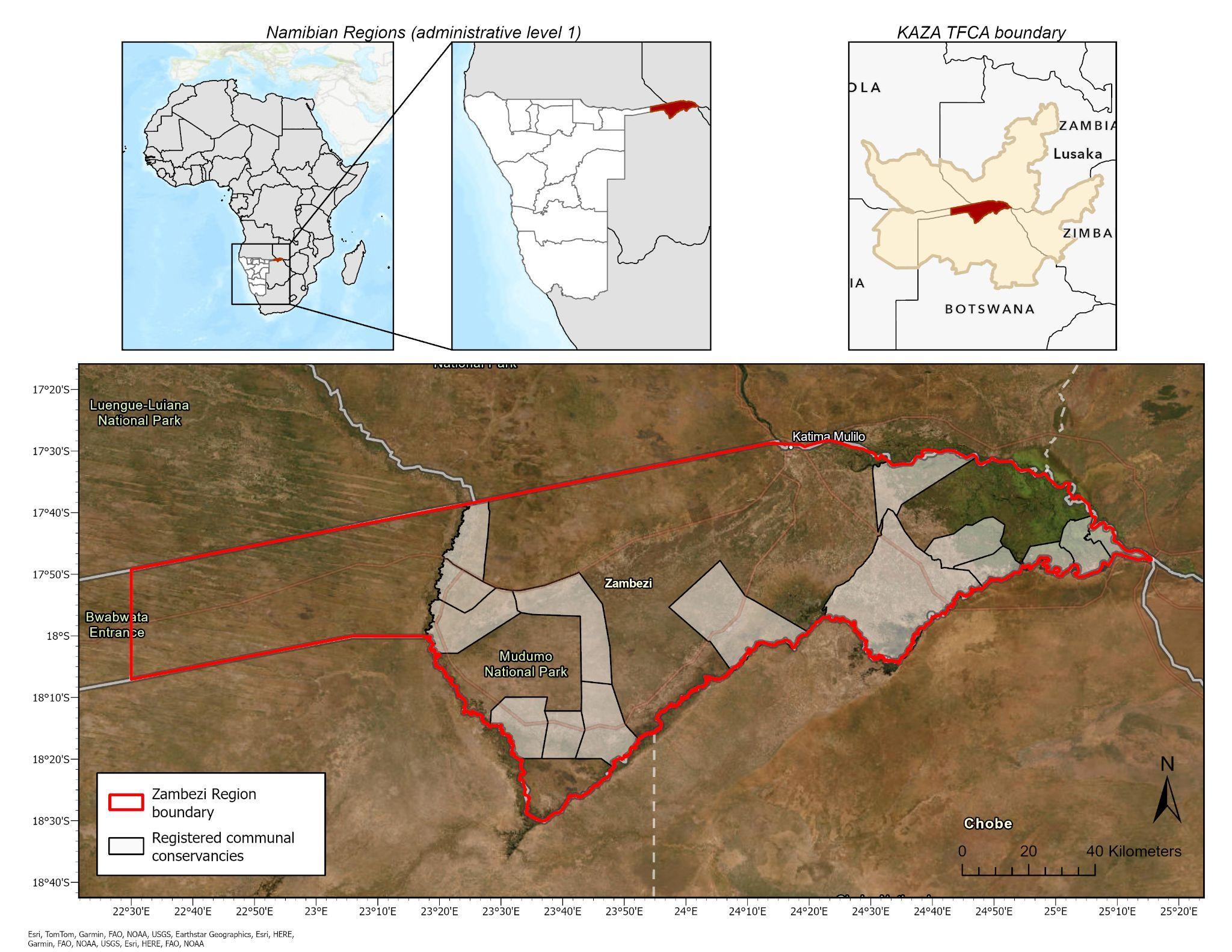


**Fig. S18.** Zambezi Region of Namibia with Namibian administrative level 1 regions (top left), the Kavango Zambezi Transfrontier Conservation Area (KAZA) boundary (top right), and all 15 registered communal conservancies within the Region’s boundary (bottom).


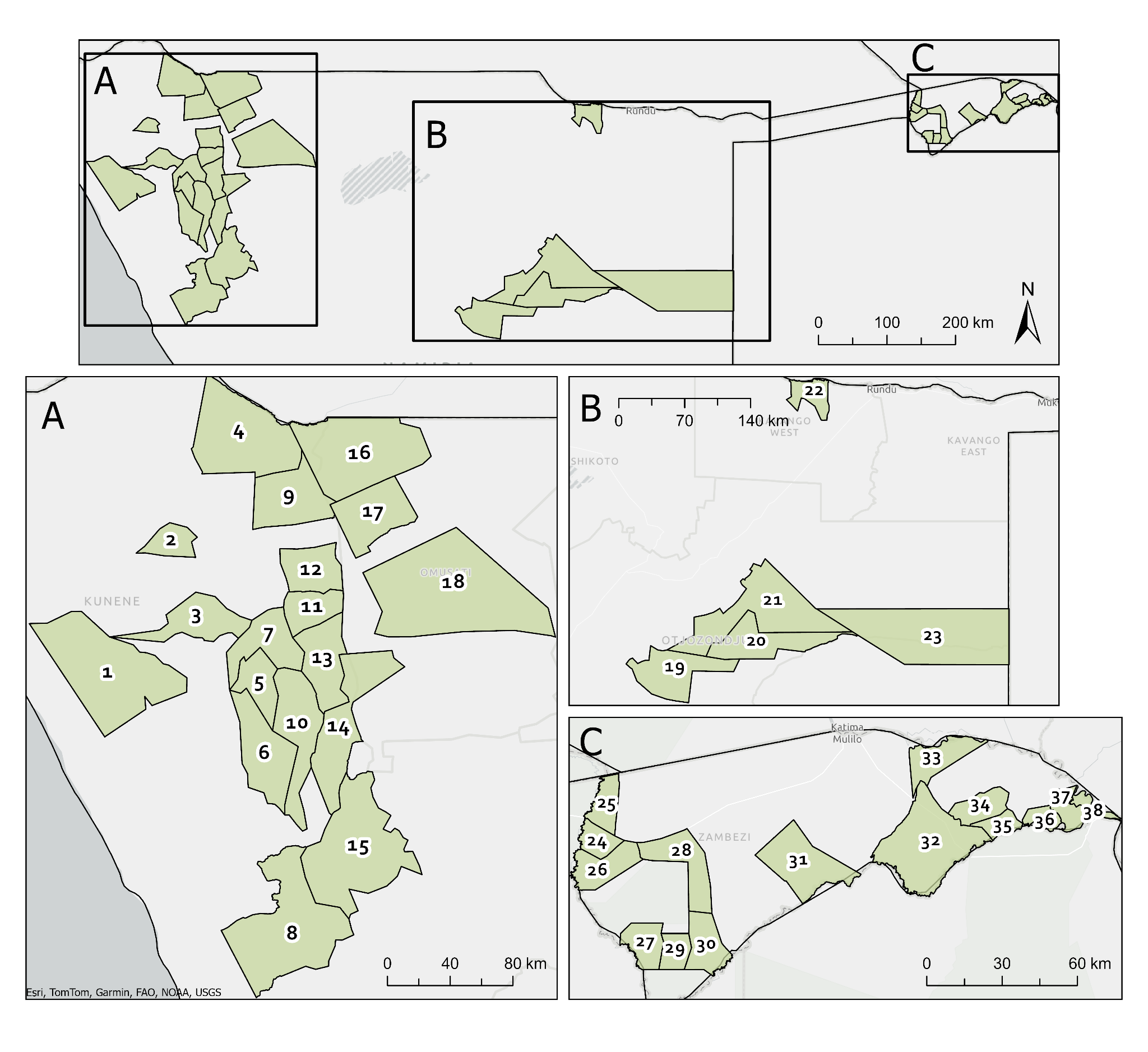


**Fig. S19.** All HEC reporting communal conservancies where the HEC data were collected. Insets and numbers for each conservancy in western (A), middle (B), and eastern (C) northern Namibia, and the names of each conservancy are listed in Table S10 below.

**Table S1**. Range and mean of conflict counts per grid cell across the whole study area, split by season.

| Season | Measure | Crop Raiding Incidents |
| --- | --- | --- |
| Wet | Range: | 0 - 75 |
|  | Mean: | 0.06759 |
| Dry | Range: | 0 - 28 |
|  | Mean: | 0.00532 |

**Table S2**. Poisson Fixed Effects coefficients and standard errors for crop raiding models. Covariates were scaled to a consistent 0-1 range before running the regression, allowing for comparable coefficients within models. * signifies a p-value of <0.05, ** refers to <0.01, and *** denotes <0.001. Count models remove within-groups without events, leading to lower numbers of observations in these models compared to the linear models.

| **Scaled Covariate** | **Crop Raiding** | |
| --- | --- | --- |
|  | **Wet Season** | **Dry Season** |
| Grid spei3 | 0.133  (0.220) | -0.283  (0.178) |
| 50km spei3 | 0.042  (0.263) | -0.465  (0.350) |
| Core spei3 | -0.289  (0.189) | **0.818***  **(0.356)** |
| Worldpop | **0.910*****  **(0.010)** | **1.256*****  **(0.182)** |
| Tree Cover | -0.014  (0.066) | -0.266  (0.138) |
| Cropland Cover | **0.149*****  **(0.037)** | 0.067  (0.105) |
| Built Cover | 0.037  (0.022) | -0.001  (0.042) |
| **Fixed Effects:** |  |  |
| Season-Year | Yes | Yes |
| Spatial Group | Yes | Yes |
| Num. Obs | 54888 | 41128 |
| Std. Errors | by: group & year | by: group & year |

**Table S3.** Negative Binomial Fixed Effects coefficients and standard errors for crop raiding models. Covariates were scaled to a consistent 0-1 range before running the regression, allowing for comparable coefficients within models. * signifies a p-value of <0.05, ** refers to <0.01, and *** denotes <0.001. Count models remove within-groups without events, leading to lower numbers of observations in these models compared to the linear models.

| **Scaled Covariate** | **Crop Raiding** | |
| --- | --- | --- |
|  | **Wet Season** | **Dry Season** |
| Grid spei3 | 0.019  (0.213) | **-0.458***  **(0.197)** |
| 50km spei3 | 0.285  (0.229) | -0.271  (0.492) |
| Core spei3 | -0.265  (0.205) | 0.548  (0.438) |
| Worldpop | **1.216*****  **(0.106)** | **1.319*****  **(0.159)** |
| Tree Cover | 0.019  (0.087) | -0.101  (0.193) |
| Cropland Cover | **0.241*****  **(0.069)** | 0.172  (0.110) |
| Built Cover | 0.096  (0.120) | 0.023  (0.044) |
| **Fixed Effects:** |  |  |
| Season-Year | Yes | Yes |
| Spatial Group | Yes | Yes |
| Num. Obs | 54888 | 41128 |
| Std. Errors | by: group & year | by: group & year |

**Table S4.** Random Effects outcomes and standard errors for crop raiding models. Covariates were scaled to a consistent 0-1 range before running the regression, allowing for comparable coefficients within models. * signifies a p-value of <0.05, ** refers to <0.01, and *** denotes <0.001.

| **Scaled Covariate** | **Wet Season^^[[1]](#footnote-1)^^** | **Dry Season** |
| --- | --- | --- |
| Intercept | 0.299 (0.174) | 0.037 (0.031) |
| Grid spei3 | -0.124 (0.083) | 0.008 (0.007) |
| 50km spei3 | 0.127 (0.192) | 0.019 (0.036) |
| Core spei3 | 0.031 (0.221) | -0.028 (0.034) |
| Worldpop | **0.266* (0.132)** | **0.050** (0.015)** |
| Tree Cover | **0.356** (0.093)** | 0.015 (0.009) |
| Cropland Cover | 0.247 (0.140) | -0.006 (0.010) |
| Built Cover | 0.016 (0.016) | 0.002 (0.002) |
| Elevation | **-0.446* (0.193)** | -0.018 (0.035) |
| Slope | **-0.381* (0.172)** | -0.021 (0.025) |
| Aspect | **0.299* (0.093)** | -0.012 (0.010) |
| Distance to rivers | -0.098 (0.216) | 0.021 (0.048) |
| Distance to roads | **0.638*** (0.158)** | **0.027* (0.009)** |
| Distance to core areas | -0.086 (0.171) | 0.021 (0.027) |
| Distance to Fences | **-0.681*** (0.111)** | **-0.035* (0.013)** |
| Distance to water | 0.000 (0.000) | **-0.000002* (0.000001)** |
| Cropland edge length | -0.001 (0.004) | -0.001 (0.001) |
| Built area edge length | **-0.023*** (0.005)** | **-0.001* (0.000)** |
| Forest edge length | **-0.014** (0.005)** | -0.001 (0.000) |

**Table S5.** Number of occurrences of HEC and background points used in each point process model.

| Season | HEC Occurrences | Background Points |
| --- | --- | --- |
| Dry | n = 692 | n = 2757 |
| Wet | n = 1811 | n = 7233 |

**Table S6.** Yearly change in crop raiding estimated from the fixed effects models, with a 95% CI interval calculated from a linear combination of errors in parentheses below. Scenarios are SSP1-RCP2.6 (‘Sustainability’), SSP2-RCP4.5 (‘Middle of the Road’), SSP3-RCP7.0 (‘Regional Rivalry’), and SSP5-RCP8.5 (‘Fossil-Fueled Development’). ‘Fossil-Fueled Development’ reports relatively low levels of land conversion and moderate population growth in the region despite high levels of emissions/warming, leading to a lower increase in HEC than other high-emissions scenarios.

| Model | SSP1-RCP 2.6  Change 2025-2085 | SSP2-RCP 4.5  Change 2025-2085 | SSP3-RCP 7.0  Change 2025-2085 | SSP5-RCP 8.5 Change 2025-2085 |
| --- | --- | --- | --- | --- |
| Wet Szn Crop Raiding | 0.431  (0.382, 0.479) | 0.695  (0.631, 0.760) | 1.04  (0.951, 1.13) | 0.502  (0.434, 0.570) |
| Dry Szn Crop Raiding | 0.031  (0.019, 0.042) | 0.048  (0.032, 0.064) | 0.073  (0.051, 0.095) | 0.029  (0.011, 0.047) |

**Table S7.** Average HEC grid-level increases in Wet Season Crop Raiding, split by conservancy and scenario. Standard errors, as derived from the Delta method (see *Materials and Methods)*, are provided in parentheses beside the projected increase values.

| Conservancy Name | Projected Wet Season Crop Raiding Change 2025-2085 | | | |
| --- | --- | --- | --- | --- |
|  | SSP1-RCP2.6 | SSP2-RCP4.5 | SSP3-RCP7.0 | SSP5-RCP8.5 |
| ≠Khoadi-//Hôas | 0.448 (0.020) | 0.614 (0.029) | 0.757 (0.037) | 0.533 (0.035) |
| Anabeb | 0.236 (0.011) | 0.317 (0.019) | 0.385 (0.025) | 0.319 (0.027) |
| Balyerwa | 0.541 (0.030) | 1.184 (0.046) | 1.469 (0.066) | 0.637 (0.034) |
| Bamunu | 0.702 (0.039) | 1.228 (0.051) | 1.968 (0.075) | 0.771 (0.045) |
| Dzoti | 0.525 (0.034) | 1.060 (0.042) | 1.625 (0.061) | 0.603 (0.040) |
| Ehi-Rovipuka | 0.450 (0.018) | 0.568 (0.026) | 0.704 (0.033) | 0.540 (0.030) |
| Impalila | 9.923 (0.385) | 12.787 (0.483) | 21.996 (0.769) | 9.645 (0.376) |
| Kabulabula | 1.108 (0.043) | 1.285 (0.048) | 1.614 (0.058) | 1.080 (0.045) |
| Kasika | 2.896 (0.112) | 3.827 (0.139) | 6.200 (0.219) | 2.803 (0.114) |
| Kunene River | 0.577 (0.027) | 0.795 (0.034) | 1.095 (0.043) | 0.658 (0.035) |
| Kwandu | 3.381 (0.126) | 4.654 (0.165) | 7.714 (0.271) | 3.283 (0.124) |
| Lusese | 1.858 (0.068) | 2.403 (0.087) | 3.557 (0.129) | 1.859 (0.072) |
| Mashi | 1.110 (0.050) | 1.827 (0.071) | 2.762 (0.010) | 1.209 (0.056) |
| Maurus Nekaro | 1.222 (0.101) | 2.625 (0.133) | 3.512 (0.158) | 1.295 (0.104) |
| Mayuni | 3.053 (0.168) | 5.630 (0.230) | 9.383 (0.352) | 2.714 (0.159) |
| Nakabolelwa | 0.784 (0.032) | 0.935 (0.036) | 1.157 (0.044) | 0.845 (0.037) |
| Okamatapati | 0.115 (0.007) | 0.170 (0.011) | 0.224 (0.015) | 0.188 (0.015) |
| Okangundumba | 0.716 (0.029) | 1.003 (0.040) | 1.277 (0.051) | 0.796 (0.039) |
| Okongoro | 1.111 (0.040) | 1.304 (0.049) | 1.562 (0.060) | 1.200 (0.049) |
| Omatendeka | 0.374 (0.016) | 0.487 (0.024) | 0.624 (0.031) | 0.461 (0.029) |
| Ombombo | 0.667 (0.031) | 0.927 (0.040) | 1.331 (0.052) | 0.748 (0.039) |
| Ombujokanguindi | 0.278 (0.012) | 0.359 (0.019) | 0.446 (0.024) | 0.367 (0.025) |
| Ondjou | 0.089 (0.006) | 0.140 (0.010) | 0.187 (0.014) | 0.159 (0.014) |
| Ongongo | 0.082 (0.005) | 0.143 (0.011) | 0.181 (0.015) | 0.170 (0.017) |
| Orupupa | 0.574 (0.023) | 0.702 (0.030) | 0.889 (0.038) | 0.663 (0.034) |
| Otjituuo | 0.113 (0.008) | 0.201 (0.013) | 0.273 (0.017) | 0.197 (0.016) |
| Otuzemba | 0.373 (0.015) | 0.481 (0.022) | 0.610 (0.028) | 0.464 (0.025) |
| Ozonahi | -0.276 (0.021) | 0.059 (0.018) | 0.237 (0.020) | -0.205 (0.029) |
| Ozondundu | 0.388 (0.016) | 0.492 (0.023) | 0.614 (0.030) | 0.477 (0.029) |
| Puros | 0.046 (0.005) | 0.094 (0.013) | 0.129 (0.020) | 0.138 (0.025) |
| Salambala | 2.417 (0.105) | 3.643 (0.139) | 4.958 (0.181) | 2.474 (0.109) |
| Sheya Shuushona | 0.310 (0.020) | 0.465 (0.025) | 0.626 (0.030) | 0.389 (0.030) |
| Sikunga | 3.861 (0.161) | 5.534 (0.203) | 11.680 (0.410) | 3.590 (0.155) |
| Sobbe | 0.044 (0.020) | 0.369 (0.023) | 0.687 (0.030) | 0.118 (0.026) |
| Torra | 0.065 (0.008) | 0.124 (0.017) | 0.161 (0.023) | 0.156 (0.029) |
| Uukolonkadhi Ruacana | 0.798 (0.068) | 1.821 (0.082) | 3.355 (0.126) | 0.832 (0.074) |
| Uukwaluudhi | 0.194 (0.021) | 0.507 (0.023) | 0.659 (0.029) | 0.270 (0.030) |
| Wuparo | 0.012 (0.054) | 0.342 (0.054) | 1.355 (0.086) | -0.063 (0.062) |

**Table S8.** Average HEC grid-level increases in Dry Season Crop Raiding, split by conservancy and scenario. Standard errors, as derived from the Delta method (see *Materials and Methods)*, are provided in parentheses beside the projected increase values.

| Conservancy Name | Projected Dry Season Crop Raiding Change 2025-2085 | | | |
| --- | --- | --- | --- | --- |
|  | SSP1-RCP2.6 | SSP2-RCP4.5 | SSP3-RCP7.0 | SSP5-RCP8.5 |
| ≠Khoadi-//Hôas | 0.032 (0.005) | 0.042 (0.008) | 0.051 (0.009) | 0.033 (0.009) |
| Anabeb | 0.016 (0.004) | 0.019 (0.006) | 0.023 (0.007) | 0.016 (0.008) |
| Balyerwa | 0.038 (0.007) | 0.086 (0.010) | 0.107 (0.014) | 0.040 (0.009) |
| Bamunu | 0.051 (0.008) | 0.089 (0.011) | 0.146 (0.016) | 0.050 (0.011) |
| Dzoti | 0.037 (0.007) | 0.076 (0.009) | 0.119 (0.013) | 0.037 (0.010) |
| Ehi-Rovipuka | 0.033 (0.005) | 0.039 (0.007) | 0.048 (0.009) | 0.033 (0.009) |
| Impalila | 0.765 (0.071) | 0.985 (0.089) | 1.697 (0.141) | 0.737 (0.069) |
| Kabulabula | 0.082 (0.008) | 0.094 (0.010) | 0.118 (0.012) | 0.074 (0.010) |
| Kasika | 0.221 (0.021) | 0.291 (0.027) | 0.473 (0.041) | 0.207 (0.023) |
| Kunene River | 0.042 (0.007) | 0.056 (0.009) | 0.077 (0.011) | 0.041 (0.010) |
| Kwandu | 0.258 (0.023) | 0.354 (0.031) | 0.591 (0.050) | 0.245 (0.024) |
| Lusese | 0.140 (0.013) | 0.180 (0.018) | 0.268 (0.025) | 0.134 (0.015) |
| Mashi | 0.082 (0.010) | 0.135 (0.014) | 0.208 (0.020) | 0.084 (0.012) |
| Maurus Nekaro | 0.091 (0.019) | 0.197 (0.026) | 0.264 (0.031) | 0.090 (0.021) |
| Mayuni | 0.232 (0.031) | 0.429 (0.043) | 0.720 (0.065) | 0.201 (0.030) |
| Nakabolelwa | 0.057 (0.007) | 0.067 (0.008) | 0.082 (0.010) | 0.056 (0.009) |
| Okamatapati | 0.006 (0.003) | 0.007 (0.005) | 0.009 (0.006) | 0.004 (0.006) |
| Okangundumba | 0.053 (0.007) | 0.072 (0.010) | 0.092 (0.012) | 0.053 (0.010) |
| Okongoro | 0.084 (0.009) | 0.095 (0.011) | 0.114 (0.014) | 0.084 (0.012) |
| Omatendeka | 0.027 (0.005) | 0.032 (0.007) | 0.041 (0.009) | 0.027 (0.008) |
| Ombombo | 0.049 (0.007) | 0.066 (0.010) | 0.096 (0.012) | 0.048 (0.010) |
| Ombujokanguindi | 0.019 (0.004) | 0.022 (0.006) | 0.028 (0.008) | 0.020 (0.008) |
| Ondjou | 0.004 (0.003) | 0.005 (0.004) | 0.007 (0.006) | 0.003 (0.006) |
| Ongongo | 0.004 (0.003) | 0.006 (0.005) | 0.007 (0.006) | 0.004 (0.007) |
| Orupupa | 0.042 (0.006) | 0.049 (0.008) | 0.062 (0.010) | 0.043 (0.009) |
| Otjituuo | 0.006 (0.003) | 0.009 (0.005) | 0.013 (0.007) | 0.005 (0.007) |
| Otuzemba | 0.027 (0.005) | 0.032 (0.007) | 0.040 (0.009) | 0.027 (0.008) |
| Ozonahi | -0.024 (0.005) | -0.002 (0.006) | 0.010 (0.007) | -0.027 (0.009) |
| Ozondundu | 0.028 (0.005) | 0.033 (0.007) | 0.041 (0.009) | 0.029 (0.008) |
| Puros | 0.001 (0.003) | 0.001 (0.005) | 0.002 (0.006) | 0.002 (0.007) |
| Salambala | 0.184 (0.020) | 0.276 (0.026) | 0.377 (0.034) | 0.182 (0.021) |
| Sheya Shuushona | 0.021 (0.006) | 0.030 (0.007) | 0.040 (0.009) | 0.020 (0.009) |
| Sikunga | 0.295 (0.030) | 0.423 (0.038) | 0.898 (0.076) | 0.268 (0.030) |
| Sobbe | -0.001 (0.005) | 0.022 (0.006) | 0.047 (0.008) | 0.000 (0.007) |
| Torra | 0.002 (0.003) | 0.004 (0.005) | 0.005 (0.006) | 0.003 (0.007) |
| Uukolonkadhi Ruacana | 0.059 (0.013) | 0.135 (0.017) | 0.251 (0.025) | 0.054 (0.016) |
| Uukwaluudhi | 0.012 (0.006) | 0.033 (0.007) | 0.043 (0.009) | 0.011 (0.009) |
| Wuparo | -0.003 (0.011) | 0.021 (0.012) | 0.099 (0.018) | -0.014 (0.014) |

**Table S9**. Summary data of conservancies from [NACSO](https://www.nacso.org.na/conservancies).

| Conservancy Name | Year of Registry | Area (km.) | Region | Reporting Date | Population Size |
| --- | --- | --- | --- | --- | --- |
| ≠Khoadi-//Hôas | 1998 | 3353.08 | Kunene | 2/7/2007 | 5629 |
| Anabeb | 2003 | 1569.99 | Kunene | 2/12/2016 | 1562 |
| Balyerwa | 2006 | 225.20 | Zambezi | 1/20/2006 | 1462 |
| Bamunu | 2011 | 555.92 | Zambezi | 1/12/2009 | 2302 |
| Dzoti | 2009 | 287.00 | Zambezi | 1/9/2009 | 2286 |
| Ehi-Rovipuka | 2001 | 1979.81 | Kunene | 9/6/2010 | 1346 |
| Impalila | 2005 | 72.50 | Zambezi | 1/30/2006 | 1000 |
| Kabulabula | 2011 | 89.00 | Zambezi | 2/3/2009 | 421 |
| Kasika | 2005 | 146.59 | Zambezi | 4/16/2009 | 1085 |
| Kunene River | 2006 | 2763.61 | Kunene | 1/31/2018 | 8499 |
| Kwandu | 1999 | 189.52 | Zambezi | 1/8/2008 | 4005 |
| Lusese | 2014 | 206.50 | Zambezi | 2/5/2010 | 1340 |
| Mashi | 2003 | 296.77 | Zambezi | 5/19/2004 | 2523 |
| Maurus Nekaro | 2017 | 1117.41 | Kavango | 3/5/2018 | 13328 |
| Mayuni | 1999 | 150.57 | Zambezi | 2/15/2004 | 2759 |
| Nakabolelwa | 2014 | 114.00 | Zambezi | 5/7/2005 | 842 |
| Okamatapati | 2005 | 3095.57 | Otjozondjupa | 12/14/2018 | 2066 |
| Okangundumba | 2003 | 1130.75 | Kunene | 1/3/2007 | 2331 |
| Okongoro | 2012 | 956.00 | Kunene | 1/27/2016 | 2230 |
| Omatendeka | 2003 | 1619.38 | Kunene | 2/6/2007 | 2939 |
| Ombombo | 2014 | 1486.76 | Kunene | 2/9/2016 | 3180 |
| Ombujokanguindi | 2012 | 1160.00 | Kunene | 6/1/2018 | 652 |
| Ondjou | 2006 | 8729.00 | Omaheke | 1/29/2019 | 3068 |
| Ongongo | 2012 | 501.32 | Kunene | 4/6/2016 | 971 |
| Orupupa | 2011 | 1234.14 | Kunene | 3/5/2007 | 1387 |
| Otjituuo | 2005 | 6132.52 | Otjozondjupa | 4/20/2020 | 5971 |
| Otuzemba | 2012 | 741.76 | Kunene | 5/2/2007 | 449 |
| Ozonahi | 2005 | 3203.53 | Otjozondjupa | 2/7/2021 | 11614 |
| Ozondundu | 2003 | 745.35 | Kunene | 9/22/2007 | 390 |
| Puros | 2000 | 3562.43 | Kunene | 5/1/2017 | 1584 |
| Salambala | 1998 | 929.79 | Zambezi | 1/30/2007 | 9193 |
| Sheya Shuushona | 2005 | 5065.57 | Omusati | 2/21/2016 | 3789 |
| Sikunga | 2009 | 286.70 | Zambezi | 4/15/2007 | 2478 |
| Sobbe | 2006 | 390.70 | Zambezi | 1/11/2009 | 1115 |
| Torra | 1998 | 3492.76 | Kunene | 1/24/2011 | 1520 |
| Uukolonkadhi Ruacana | 2005 | 2992.91 | Omusati | 2/16/2016 | 37712 |
| Uukwaluudhi | 2003 | 1436.78 | Omusati | 4/10/2016 | 1088 |
| Wuparo | 1999 | 147.6 | Zambezi | 2/14/2006 | 987 |

# Table S10. HEC reporting communal conservancies associated with Figure S19.

| Conservancy Name | Number in Fig. S19 |
| --- | --- |
| Puros | 1 |
| Ongongo | 2 |
| Ombujokanguindi | 3 |
| Kunene River | 4 |
| Ozondundu | 5 |
| Anabeb | 6 |
| Okangundumba | 7 |
| Torra | 8 |
| Ombombo | 9 |
| Omatendeka | 10 |
| Otuzemba | 11 |
| Okongoro | 12 |
| Orupupa | 13 |
| Ehi-Rovipuka | 14 |
| ≠Khoadi-//Hôas | 15 |
| Uukolonkadhi Ruacana | 16 |
| Uukwaluudhi | 17 |
| Sheya Shuushona | 18 |
| Ozonahi | 19 |
| Okamatapati | 20 |
| Otjituuo | 21 |
| Maurus Nekaro | 22 |
| Ondjou | 23 |
| Mayuni | 24 |
| Kwandu | 25 |
| Mashi | 26 |
| Balyerwa | 27 |
| Sobbe | 28 |
| Wuparo | 29 |
| Dzoti | 30 |
| Bamunu | 31 |
| Salambala | 32 |
| Sikunga | 33 |
| Lusese | 34 |
| Nakabolelwa | 35 |
| Kabulabula | 36 |
| Kasika | 37 |
| Impalila | 38 |

1. The wet season crop raiding random effects model failed the Hausman test, meaning that it is not appropriate for causal inference as there is unobserved variation that impacts the outcome across spatial and time groups. However, we report results here to allow for some comparison with the point process models. The dry season model passed the Hausman test. [↑](#footnote-ref-1)
